# Microbially induced calcite precipitation (MICP): mechanisms, engineering applications, and pathway to field-scale deployment — a comprehensive review

**DOI:** 10.3389/fmicb.2026.1928040

**Published:** 2026-08-27

**Authors:** Tao He, Jiaxing Chen, Xiyan Lou

**Affiliations:** Shangqiu Normal University, Shangqiu, Henan, China

**Keywords:** biocementation, field-scale deployment, heavy metal immobilization, liquefaction, MICP, self-healing concrete, soil stabilization, *Sporosarcina pasteurii*

## Abstract

Over the last twenty years, there has been a great amount of multidisciplinary interest in Microbially Induced Calcite Precipitation (MICP) as a truly sustainable solution to ground treatment using Portland cement. The process utilizes the urease activity of ureolytic bacteria, most commonly *Sporosarcina pasteurii* to convert soluble urea and calcium to solid calcite in the pore space of granular media, providing cementation, impermeability reduction and contaminant sequestration without excavation or heat. This review compiles over sixty publications covering all aspects of MICP research, ranging from the enzymological chemistry of urease to the documented field trials of over 100 m^3^ of treated volume, to the thermodynamics of calcium carbonate polymorphism. Unconfined compressive strengths of over 10 MPa have been reached in laboratory columns and wind erosion reduction of 95% has been observed in field tests, while the efficiency of heavy metal removal can reach up to 99% for lead in aqueous laboratory systems, though removal efficiencies in soil matrices are typically lower and highly dependent on metal concentration, soil type, and treatment conditions. The self healing concrete application is the most advanced toward commercial maturity, and there are products available from Basilisk, Evonik and BioMason. Ground improvement, liquefaction mitigation, and coastal erosion control are other geotechnical applications at Technology Readiness Levels 5 to 7, with the challenges of uniformity of treatment in heterogeneous soils, management of the by-product of ammonium, bacterial transport at depth, and competitiveness with conventional grouting. New developments such as non-ureolytic microbial pathways, fiber and biochar reinforced hybrid biocementation, 3D bio-printing of living mineral structures, and process optimization via machine learning with R^2^ > 0,95, are also being explored. The review closes with a candid critique of the gaps in the research, especially long-term durability information, scale-specific cost analyses, and standardized monitoring protocols, which will decide whether or not MICP becomes a successful laboratory-based concept that moves into a common engineering tool.

## Graphical abstract

**Figure:**
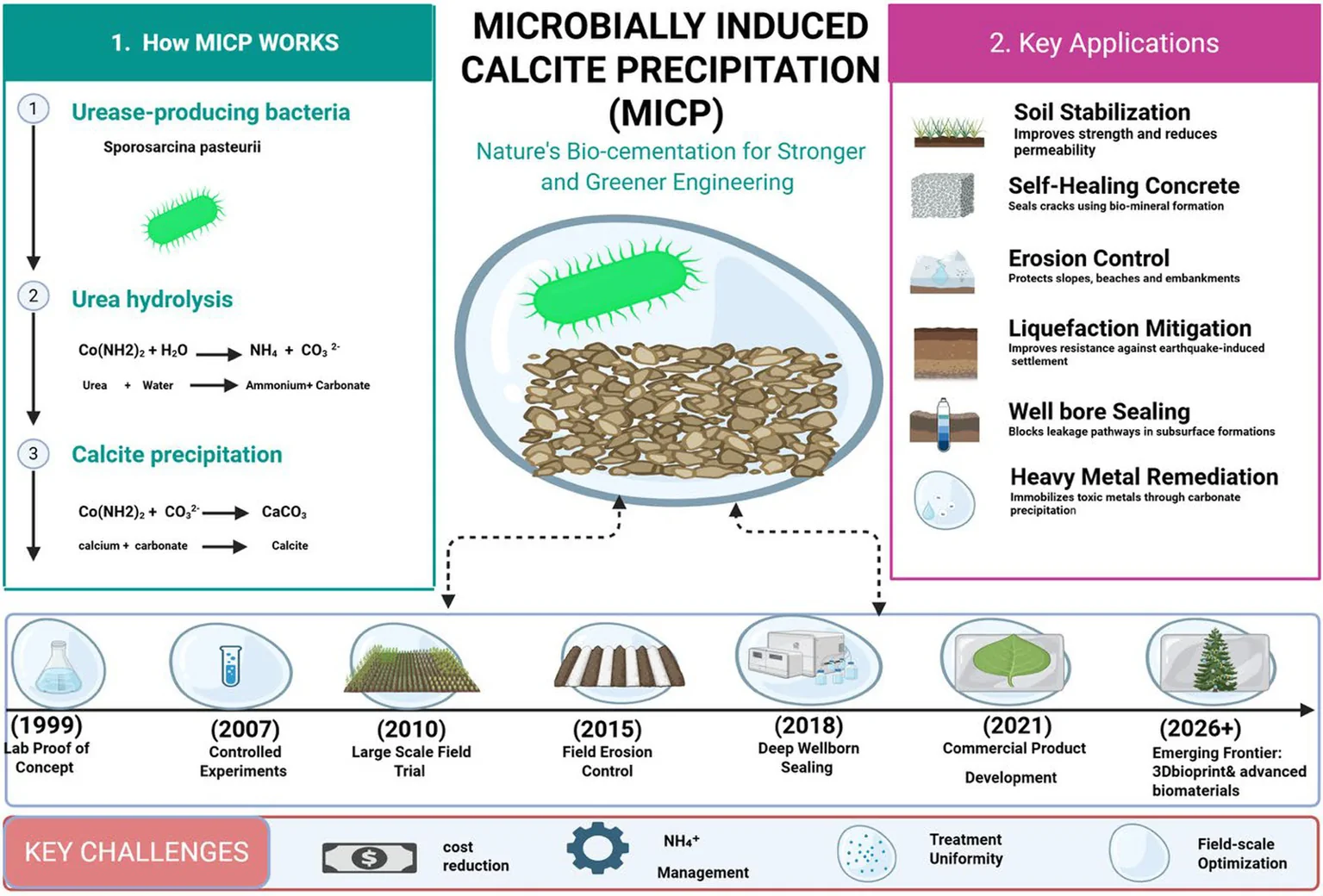
Schematic overview of the MICP process: bacterial ureolysis drives CaCO₃ precipitation in soil pores, underpinning six engineering applications spanning TRL 2–9. A 1999–2026 deployment timeline tracks the technology’s progression from laboratory proof-of-concept to commercial self-healing concrete products, highlighting remaining barriers to large-scale field adoption.

## 1 Introduction

Civil construction represents a significant and increasing portion of emissions of global carbon, especially from the Portland cement industry which accounts for an estimated 7–8% of anthropogenic CO₂ emissions annually. The need for ground improvement, foundation stabilization and infrastructure repair will continue to grow as urbanisation progresses, especially in Asia, Africa and Latin America; and the need to develop alternatives to carbon-intensive methods will be growing. In this context, the idea of directly using the metabolic processes of microorganisms to form mineral cement in the pores of soil has received continuing and growing interest since it was first distinctly expressed in the late 1990s (Stocks-Fischer et al., 1999; Fujita et al., 2000; Mitchell and Santamarina, 2005).

Conceptually, this effort is called Microbially Induced Calcite Precipitation (MICP) and is very elegant. Some bacteria, when provided with a soluble calcium salt and urea, break the urea down into carbonate ions, thereby increasing the pH of the surrounding environment which leads to the precipitation of calcium carbonate at points of contact with the grain and in the pores (Hammes et al., 2003; DeJong et al., 2006). These mineral bridges can significantly improve shear strength, decrease permeability, bind heavy metals, and seal cracks, without the use of excavation, vibration or chemical toxicity. It’s true to its claims: the product contains urea, one of the most plentiful commodity chemicals available on Earth; the common bacterial strains used are not pathogenic; and the mineral product is geochemically stable and ecologically benign.

Over 20 years of research and several published peer reviewed articles now address the study of MICPfrom many angles, including: enzymology geomicrobiology, soil mechanics, concrete durability, and environmental remediation. Key milestones include the first unambiguous laboratory demonstration of MICP-induced strength gain in sand columns by DeJong et al. (2006); the large-scale 100 m^3^ field trial in the Netherlands by van Paassen et al. (2010); the introduction of self-healing concrete based on encapsulated bacterial spores by Jonkers et al. (2010); and the development of centrifuge-scale evidence for liquefaction resistance improvement by Kamalzare et al. (2013). More recently, Xiao et al. (2025) conducted a bibliometric analysis of 5,373 MICP publications, identifying amorphous calcium carbonate and biofilm interfaces as emerging research frontiers.

Despite this body of work, the consensus view among active researchers is that MICP remains a largely pre-commercial technology in geotechnical applications, held back by practical challenges that laboratory columns by their very nature systematically understate. This review is structured to address that gap directly: it begins with the biochemistry and microbiology, then covers governing factors, the full range of engineering applications with quantitative performance data (Section 4), injection and delivery strategies, the documented field-scale experience and the practical barriers it has exposed, and the most significant recent advances including commercialization status. Section 8 identifies the research priorities whose resolution would most consequentially advance the field.

## 2 Biochemical mechanisms of MICP

An understanding of the biochemistry of MICP is not only a subject of academic interest, it is an important aspect of the process itself. 79 describes design decisions for process, applicability and nature of unwanted side products. The following sections draw on a literature spanning foundational geomicrobiology (Stocks-Fischer et al., 1999; Hammes et al., 2003) through to current molecular biology of the urease enzyme (Krajewska, 2018; Haystead et al., 2024; Zhu et al., 2024).

### 2.1 The ureolysis reaction

Urease (urea amidohydrolase; EC 3.5.1.5) catalyzes the hydrolysis of urea according to the overall stoichiometry first applied to soil biocement context by Stocks-Fischer et al. (1999) and thermodynamically analyzed in depth by Hammes et al. (2003)

$$\mathrm{CO}{\left(NH_{2}\right)}_{2}+2H_{2}O\overset{\text{urease}}{\to }2NH_{4}^{+}+CO_{3}^{2-}$$

One mole of urea yields two moles of ammonium and one mole of carbonate. The net consumption of protons elevates local pH to approximately 8.3–9.3, creating conditions of high carbonate supersaturation. In the presence of dissolved calcium ions:

$$Ca^{2+}+C{O_{3}}^{2-}\to \text{CaCO}3↓$$

The net biocementation reaction therefore reads:

$$\mathrm{CO}{\left(NH_{2}\right)}_{2}+2H_{2}O+Ca^{2+}\overset{\text{bacteria}}{\to }\mathrm{CaC}O_{3}↓+2NH_{4}^{+}$$

**Figure 1:**
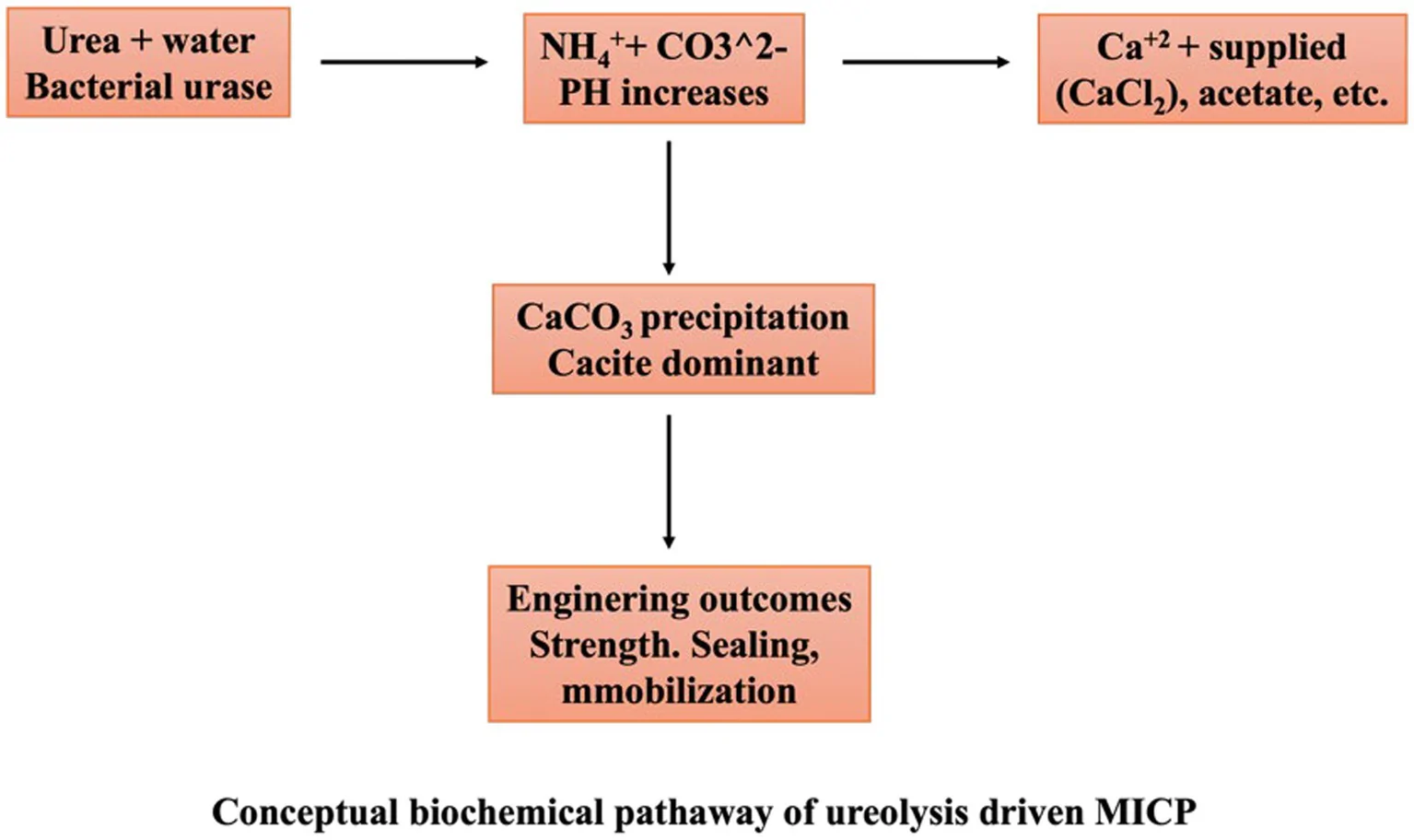
Conceptual biochemical pathway of ureolysis-driven MICP, from enzymatic urea hydrolysis through carbonate ion generation and calcium carbonate precipitation to engineering outcomes.

Hammes et al. (2003) provided a detailed thermodynamic analysis of the individual equilibria, showing that the calcium carbonate saturation index is particularly sensitive to temperature and initial urea concentration. Precipitation is best at pH 8.3–9.2, and will not occur below pH carbonate protonnation prevents supersaturation and the rate of precipitation becomes practically zero (Fujita et al., 2004) extended this to demonstrate that trace divalent cations, such as Sr^2+^ the coprecipitation of and Ba^2+^ with calcite through isomorphous substitution was observed, which is the basis of this in applications for removal of heavy metals (Figure 1).

### 2.2 Key microorganisms and cultivation requirements

The reaction of ureolysis can be catalysed by any organism which contains urease, however, the research conducted in applied MICP has focused on a few species that possess both a high urease activity and cultivation characteristics. *Sporosarcina pasteurii* is now the preferred model organism as it is an obligate aerobic and alkaliphile that has urease activity that substantially exceeds that of other species that are commonly tested, and withstands the high pH/high urea conditions typical of the MICP protocols (Stocks-Fischer et al., 1999; DeJong et al., 2006). In a systematic study of the nutrient requirements of *S. pasteurii* in defined media, Lapierre et al. (2020) showed that there is a significant over-supply of essential nutrients for *S. pasteurii* in standard rich-broth cultivation conditions compared to what could be supplied in the field subsurface environments, a crucial finding for scaling predictions. The urease activity of *S. pasteurii* is sensitive to salinity, temperature, and dissolved oxygen (DO) as documented by Mortensen et al. (2011), which defines the design envelope for MICP treatment to be reliable.

It is not only *S. pasteurii* that has been explored, but also a variety of *Bacillus* species, notably *B. subtilis, B. megaterium, B. sphaericus and B. cereus*, which because of their endospore-forming capacity are interesting for their use in applications where long term bacterial dormancy is required in harsh environments like concrete (Jonkers et al., 2010; Van Tittelboom et al., 2010; Wiktor and Jonkers, 2011). As demonstrated by Achal et al. (2011), *B. pasteurii* CT-5 was able to enhance the compressive strength of cement mortar, whereas while Wang et al. (2012), Wang et al. (2014) devised silica gel and hydrogel encapsulation strategies to shield spores from the highly alkaline concrete matrix. *Lysinibacillus sphaericus* is gaining increasing interest due to its ability to work at lower temperatures and its tolerance of seawater use conditions (Zhu et al., 2024).The main bacteria identified in the literature and relevant to MICP are summarized in Table 1.

**Table 1:** Microorganisms and reporting status of urreases measurement.

Organism/Pathway	Strain	Assay and normalization	Substrate/pH/temperature	Replicate statistic	Design relevance	References
*Sporosarcina pasteurii*; ureolysis	Multiple study specific strains	Whole-cell kinetics reported by Lauchnor et al. (2015)	Urea ≤330 mmol L^−1^ for first-order fit; pH 6–9	n and SD unavailable in reviewed source set	Use site relevant whole-cell rate and retained cell density	Dejong et al. (2006), Stocks-Fischer et al. (1999), Lauchnor et al. (2015)
*Bacillus subtilis*; ureolytic/non-ureolytic reports	Study-specific; recombinant Strain B (WB 600/pHT254-UreABCEFGD)	Gene vector construction; PCR validation; urease gene cluster expression	37 °C; optimal cementation solution: 1.5 M; bacteria-to-cementation ratio 1:1.25	OD600 measurements; triplicate experiments	Validate strain, assay, and matrix before design use; recombinant strain showed 44.6% higher urease activity than *B. pasteurii*	Zhang et al. (2026)
*Bacillus megaterium*; ureolysis	Study-specific; MTCC 2412; ATCC 14581	Dual urease and carbonic anhydrase capabilities; FTIR, EDX, SEM characterization	30 °C; pH conditions; CO₂ levels >470 × atmospheric activate carbonic anhydrase pathway	94.1% ± 0.1% of CaCO₃ from CO₂ mineralization	Requires strain- and matrix-specific validation; favors CO₂ mineralization over ureolytic pathway at elevated CO₂	Cappa et al. (2025), Dhriyan et al. (2025)
*Lysinibacillus sphaericus*; ureolysis	PG22 (marine isolate); LMG 22257	Ureolytic activity; TGA, XRD, ESEM-EDX analyses	30 °C; 1,600 ppm Pb(NO₃)₂ tolerance; CaCl₂·2H₂O 0.3–1.0 M	61.7 g/L CaCO₃; 100% Pb removal (98% biomineralized)	Potential harsh-environment use; marine-compatible; endospore-forming; effective for metal bioremediation	Joo et al. (2025), Vitale et al. (2025)
*Bacillus cereus*; biostimulation context	QBB4, QBB5 (Qatari isolates); MTCC 430	Urease production; biofilm formation	30–42 °C; pH 7.0–8.0; calcareous soils with high carbonate and alkalinity	16% CaCO₃ increase in field conditions; 7% increase in soil stability	Field use requires biosafety and community assessment; indigenous strains adapted to harsh conditions	Dhami et al. (2013), Oualha et al. (2020)
*Bacillus alkalinitrilicus*; related product claims	Encapsulated in superabsorbent polymer (SAP)	Endospore survival; germination and growth kinetics; microcalorimetry	Aerobic incubation; crosslinked acrylamide-based SAP	~80% endospore germination; up to 0.3 g CaCO₃/g desiccated SAP	Requires product-specific independent evidence; oxygen limitation affects precipitation rates	Nielsen et al. (2020)
*Pararhodobacter* sp.; denitrification	*Pararhodobacter daqingensis* (syn. *Plastorhodobacter daqingensis*)	Non-ureolytic denitrification pathway	Anaerobic; exact window unavailable	Taxonomy confirmed	Select by pathway and electron-acceptor requirements; alternative to ureolytic MICP	Xie et al. (2015)

Analytical interpretation. The table showes that cross organism ranking is presently unsupported because assay normalization and replication were not consistently reported. For enginering design, a source specific whole cell rate measured under relevant chemistry is more defensible than transferring a qualitative “high moderate” label.

### 2.3 Urease enzyme: structure, kinetics, and genetic regulation

The urease enzyme is a heterotrimer, where each subunit type is coded by a structural gene, with urease metallocentre assembly requiring four accessory proteins, ureD, ureE, ureF, and ureG, which are responsible for nickel insertion (Zhu et al., 2024). Active sites are structured to include a binuclear nickel center that is a catalyst for activating urea for nucleophilic attack. Krajewska (2018) reviewed the urease kinetics and thermodynamics, listing Michaelis–Menten constants for different species and showed that the affinity of the enzyme for urea rises significantly with pH from 7 to 9, with the highest affinity found around pH 8.3–9.0.

For *S. pasteurii*, purified enzyme Km values of 26–42 mM and Vmax of 1.7–3.6 mM min^−1^ mg-protein^−1^ have been reported (Castro-Alonso et al., 2019; Lapierre et al., 2020). Whole-cell kinetics are systematically lower, reflecting membrane mass-transfer resistance and product inhibition by accumulating NH₄^+^ a distinction that matters considerably for process models that are calibrated on purified enzyme data and then applied to column or field experiments (Hammes et al., 2003; Krajewska, 2018).

A detailed study by Lauchnor et al. (2015) demonstrated the quantitative kinetic parameters for whole-cell ureolysis by *S. pasteurii* and demonstrated that kinetic models developed using purified urease do not apply for the whole-cell ureolysis. When used for whole cells, the Michaelis–Menten model has a half-saturation coefficient Km = 305 mmol L^−1^ and a maximum rate Vmax = 200 mmol L^−1^ h^−1^. The model of the first order with the parameter k₁ = 0.35 h^−1^, however, showed better regression coefficient (R^2^ = 0.99) for urea concentrations up to 330 mmol L^−1^. The cell dependent rate constant for ureolysis was found to be 6.4 × 10^−9^ mmol CFU^−1^ h^−1^ and was linear with the cell concentration (1 × 10^7^–2 × 10^8^ CFU mL^−1^). Most importantly, even at high concentrations of ammonium (up to 0.19 mol L-1) and at a pH range of 6–9, the ureolysis rate did not significantly differ, meaning that effects of NH4^+^ inhibition and pH are not required for kinetic modeling of ureolysis for whole-cell systems at such concentrations. However, the purified urease has Km values of 26–42 mM, which are an order of magnitude lower, indicating that there is no mass transfer resistance, and no membrane permeability limitations. This distinction has engineering implications: reaction rates for purified-enzyme kinetics would systematically overestimate for field-scale MICP processes, and treatment uniformity would be mispredicted.

Molecular studies have recently helped to understand the regulation of urease gene expression in *S. pasteurii*. Pei et al. (2025) discovered for the first time that the urease of *S. pasteurii* contains a double operon, operon 1 consisting of ureA, ureB, ureC, ureE and ureF genes and operon 2 consisting of ureG and ureD genes. This discovery gives important insights into the regulation of urease expression which will enable more efficient and controllable mineralization applications. The regulation of expression is inducible and not constitutive that means that the transcription of urease is enhanced when environmental stressors like hydrocarbons are present. In response to nitrogen deficiency, this adaptation mechanism is a upregulation of the ureC gene per cell that results in ureolysis rates that are not inhibited by intracellular urease. In the field context, this means that, biostimulation strategies that induce nitrogen-limiting conditions can induce an over-activation of the urease gene and hasten the onset of MICP, versus strategies based on the constant expression of a gene can have a lower performance (Comadran-Casas et al., 2026). Strains resistant to 35 g L-1 NaCl and pH 12 have been developed in the laboratory (Liu J. et al., 2025), but their persistence in the field, biosafety, and mineralization performance need to be evaluated. In addition, studies on temperature adaptation have revealed that urease activity drops by almost 90% when the temperature is reduced to 4 °C, with the downregulation of ureABCEF and slight upregulation of ureGD. The presence of MICP at 4 °C, despite the decrease in bacterial growth and enzymatic activity, is an important factor for cold-region applications, especially for calcite crystal polymorph (Lee, 2025) (Table 2).

**Table 2:** Quantitative linkage between kinetic mechanisms and enginering design variables.

Microscale mechanism	Quantitative parameter	Engineering design variable	Implication for *In Situ* vs. *Ex Situ* design	References
Whole-cell urea hydrolysis kinetics	Km = 305 mmol L^−1^; first-order k₁ = 0.35 h^−1^ (urea ≤330 mM)	Injection frequency; retention time	In situ: Lower k₁ requires longer retention times or higher cell densities; Ex situ: Shorter retention possible with high cell density (≥2 × 10^8^ CFU mL^−1^)	Lauchnor et al. (2015)
Cell density dependence	6.4 × 10^−9^ mmol CFU^−1^ h^−1^	Cell density target; flow rate	In situ: Max cell density limited by injection logistics; low-density bioaugmentation requires extended treatment cycles; Ex situ: Cell density can be optimized at cultivation stage	Lauchnor et al. (2015)
NH₄^+^ insensitivity (up to 0.19 M)	No rate inhibition observed	By-product management strategy	In situ: NH₄^+^ accumulation does not slow reaction; treatment can proceed without rinsing until stoichiometric limit reached; Ex situ: End-of-cycle NH₄^+^ removal needed for regulatory compliance	Lauchnor et al. (2015)
pH insensitivity (6–9)	No rate inhibition observed	Chemistry of injection solutions	In situ: pH fluctuations in heterogeneous soils do not strongly affect ureolysis rate; Ex situ: Simplified pH control reduces operational complexity	Lauchnor et al. (2015)
Double operon structure/inductive regulation	*ureABCEF* + *ureGD* operons; up-regulation in N-limited conditions	Biostimulation strategy	In situ: Nitrogen limitation can induce urease expression, potentially enhancing biostimulation; Ex situ: Culture conditions can be designed to induce expression before injection	Pei et al. (2025)
Cold adaptation (4 °C)	90% urease activity reduction; calcite polymorph shift	Season/temperature selection	In situ: MICP feasible at low temperatures but slower; calcite polymorph may affect long-term durability; Ex situ: Temperature control critical for maintaining activity	Pei et al. (2025)
Treatment radius/Well spacing	Microbial transport limited to 1–3 m from injection point	Well spacing; injection pressure	In situ: Wells spaced >2 × treatment radius create untreated zones; 2-, 3-, and 4-well configurations with 1 m spacing optimized for uniform CaCO₃ distribution in numerical models	Sang et al. (2023)

### 2.4 Crystal nucleation, polymorphism, and EPS

Calcium carbonate precipitates in three principal anhydrous polymorphs under ambient conditions. Calcite (rhombohedral) is thermodynamically stable and dominates products from *S. pasteurii* in standard MICP experiments. Aragonite (orthorhombic) is favored at elevated temperatures or in magnesium-rich solutions. Vaterite (hexagonal) is the kinetically favored, thermodynamically metastable polymorph. Haystead et al. (2024) showed that high-EPS-producing *Bacillus subtilis* strains generate predominantly vaterite, stabilized by EPS functional groups particularly carboxylic and hydroxyl moieties hat adsorb onto vaterite faces and inhibit its transformation to calcite. Qiu et al. (2025) reported mixed vaterite-aragonite structures in engineered microparticle systems, illustrating that polymorph selection remains an active research frontier with implications for long-term mechanical performance of MICP-treated soils (Haystead et al., 2024).

The spatial relationship between bacterial cells, biofilm, and the precipitating mineral deserves emphasis. Dhami et al. (2013) reviewed biomineralization mechanisms across biology and engineering, noting that in MICP the cell surface acts as the primary nucleation substrate: the net negative charge of the cell wall under alkaline conditions concentrates Ca^2+^ ions, and EPS provides a structured template for oriented crystal growth. Any process model that treats calcite precipitation purely as bulk geochemical crystallization will therefore mispredict the spatial distribution of the mineral and, consequently, the mechanical contribution per gram of calcite deposited (Haystead et al., 2024).

### 2.5 Alternative biomineralization pathways

Ureolysis dominates the applied literature because it is fast and controllable, but it is not the only microbially mediated route to CaCO₃ precipitation, and its alternatives are attracting growing attention because they avoid the ammonium by-product that complicates field application. O’Donnell et al. (2017) introduced denitrification-based MICP, demonstrating that the alkalinity generated by anaerobic nitrate reduction can drive carbonate precipitation in oxygen-limited environments deep soil horizons, concrete crack interiors where ureolysis is impractical. Jain et al. (2021) further argued from a pathway comparison that denitrification may be the most field-suitable alternative to ureolysis for subsurface biocementation, particularly because it is not inhibited by low oxygen availability. Omoregie et al. (2025) developed non-ureolytic concrete self-healing systems using pathways that entirely avoid NH₃ generation, with concrete crack healing results comparable to ureolysis-based controls. Hemayati et al. (2023) demonstrated that non-ureolytic *Bacillus subtilis* and *B. amyloliquefaciens* can precipitate carbonate using calcium formate, with the formate-based approach outperforming acetate for wind-erosion control while generating no ammonium.

Although ureolysis is the most rapid and well-documented pathway, other microbial pathways via formate and urease-based EICP and formate-dehydrogenase EICP may alleviate specific limitations. Denitrification is anaerobic, does not require ammonium, is slower, but can produce gas and nitrogen-management problems. Hemayati et al. (2023) has shown a microbial carbonate precipitation method to suppress wind erosion that is not ureolytic. There is a significant EICP literature for plant urease and a non-ureolytic EICP route using formate-dehydrogenase was developed and scaled-up in the laboratory by Deylaghian et al. (2025). EICP is enzyme induced and not microbial metabolism, and is only provided for comparison.

Despite these qualitative advantages, the selection of an appropriate MICP pathaway requires carefull balancing of reaction kinetics, environmental conditions, and by products constraints. Quantitative data for pathway performance are essential for engineering design, yet most alternative pathways remain poorly characterized in terms of precipitation rates, operational windows, and field scale validation. To support pathway selection, Table 3, summarizes available quantitative evidences.

**Table 3:** Comparatively pathway selection matrix for MICP applications (using ureolysis as baseline).

Parameter	Ureolysis (U-MICP)	Denitrification (D-MICP)	Formate-based (Microbial)	FDH-EICP (Enzymatic)	References
Precipitation rate	~15 × faster than D-MICP; precipitates 2–5 × more CaCO₃ in days vs. weeks/months	Up to 0.26 w%/day; high conversion ratio	91.4% ± 1.6% calcium removal (methane-amended)	31 × strength increase after 5 cycles	Ganendra et al. (2014), Abdolvand et al. (2026)
CaCO₃ yield/Removal	High (~1 mol CaCO₃ per mol urea)	Lower than ureolysis; partially compensated by N₂ gas desaturation	0.67 ± 0.03 g CaCO₃/g Ca(CHOOH)₂	1.89% CaCO₃ after 5 cycles	Ganendra et al. (2014), Abdolvand et al. (2026)
pH optimum	8.3–9.3	~7–9 (ideal: pH = 9)	Increases during process; ~6.6–6.9 initial	7.6	Abdolvand et al. (2026), Mou et al. (2026)
Temperature optimum	20–37 °C	~30 °C	28 °C (*M. parvus*)	37 °C; >90% efficiency up to 50 °C	Ganendra et al. (2014), Pham (2017), Abdolvand et al. (2026)
O₂ requirement	Aerobic/anaerobic	Strictly anaerobic (DO < 0.2 mg/L)	Aerobic (methane-amended)	O₂-independent (non-living enzyme)	Ganendra et al. (2014), Abdolvand et al. (2026), Deylaghian et al. (2025)
By-products	2 mol NH₄^+^ per mol urea; harmful	N₂ gas (non-toxic if complete); N₂O risk (265 × CO₂ eq) if incomplete	No ureolytic NH₄^+^	No ammonium by-products	Ganendra et al. (2014), Abdolvand et al. (2026), Deylaghian et al. (2025)
Validated depth	100 m^3^ field trial; wellbore sealing at 310–700 m	Laboratory/centrifuge; in-situ groundwater potential	Laboratory-scale only	Laboratory-scale only	Abdolvand et al. (2026)
Key advantage	Most rapid, well-characterized, extensive field evidence	Native bacteria; no toxic byproducts (if complete); anaerobic compatibility	No NH₄^+^ production	No living cells; good thermal stability	Ganendra et al. (2014), Abdolvand et al. (2026), Deylaghian et al. (2025)
Key limitation	NH₄^+^ management; exogenous cultivation often required	Slower rate; risk of N₂O accumulation; O₂ sensitivity	Rate/data limited; requires specific organics	Early-stage; scale not validated	Abdolvand et al. (2026), Deylaghian et al. (2025)
Estimated TRL	5–9 (application-dependent)	4–5	3–4	3–4	Deylaghian et al. (2025)

A substantial parallel literature has developed around Enzyme-Induced Carbonate Precipitation (EICP), which uses plant-derived urease (typically from jack bean) rather than microbial urease. EICP avoids the complexities of bacterial cultivation and transport, offering a simpler treatment protocol with lower biological regulation requirements (Neupane et al., 2013; Hamdan et al., 2017). However, EICP faces its own limitations: the enzyme is more sensitive to environmental conditions, has a shorter active lifespan, and produces less uniform cementation in heterogeneous soils (Ahenkorah et al., 2024). Recent comparative studies have shown that MICP generally outperforms EICP in terms of long-term durability and strength retention under environmental stress (Jedrzejko et al., 2025), though the choice between approaches remains application-specific.”

## 3 Factors governing MICP treatment efficiency

Translating the MICP reaction from a beaker to a field treatment zone requires understanding how a set of interacting parameters modulate the quantity, spatial distribution, and mechanical contribution of precipitated calcite. Several of these have been systematically studied; others remain incompletely characterized (Mortensen et al., 2011; Al Qabany et al., 2012; Cheng et al., 2013; Mujah et al., 2017; Zhu et al., 2024).

Urea and calcium concentration. Higher concentrations of urea and CaCl₂ generate more calcite per injection cycle but also risk rapid pore clogging near injection points, creating non-uniform treatment profiles that are particularly problematic at field scale (Al Qabany et al., 2012). After performing a systematic study on the effects of chemical concentrations, Al Qabany et al. (2012) determined that, although the total content of calcite after several cycles was similar, the distribution of calcite in dilute concentrations (0.1–0.25 M urea) was more uniform than in the concentrated concentrations (0.5–1.0 M). The near 1:1 ratio of urea to calcium maximizes the conversion efficiency, and minimizes by-product buildup (Zhu et al., 2024).

Temperature. The activity of urease doubles approximately for every 10 °C rise in temperature between 10 °C and 37 °C, which means that it will naturally be slower in the field at a cold temperature than it would be in the lab at 20–25 °C (Mortensen et al., 2011). Finally, temperature can influence the polymorph: aragonite is more stable than calcite at lower temperatures, which could be advantageous for long-term mechanical stability (Haystead et al., 2024).

The pore structure and grain size of soils. The most favorable sands for MICP are coarser and well-graded: this means that the pores are large enough that bacteria cells can flow freely. Smaller materials (D10 < approx 0.1 mm) can concentrate bacteria near injection points, resulting in very strong spatial gradients of treatment intensity (*S. pasteurii* cells 1–3 μm) (Cheng et al., 2013). In a variation of the percolation method, the ‘surface percolation’ technique was introduced specifically for unsaturated coarse sands, which allows for the more uniform treatment of the sand by using a gravity-driven downward flow without the need for injection pressure (Cheng et al., 2013).

**Figure 2:**
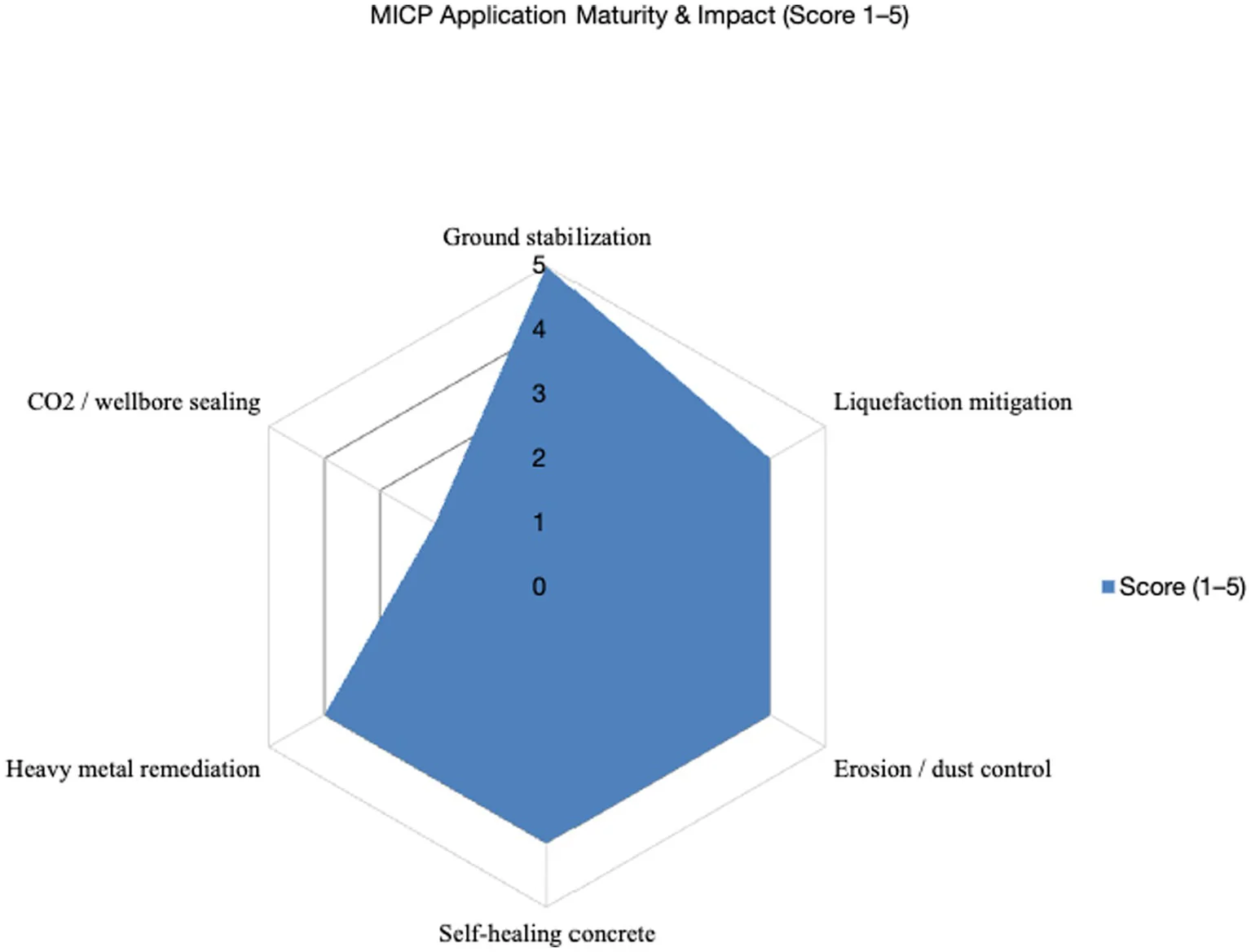
Relative maturity and engineering impact of MICP across major application domains, assessed on a 1–5 scale compiled from the reviewed literature. Ground stabilization and self-healing concrete show the highest combined scores. Scores reflect number of published studies, strength of quantitative evidence, and progress toward field deployment.

The number of cycles and treatment time. The unconfined compressive strength is power-law related to calcite content (in % of dry soil mass): the highest gain in strength with calcite deposit is in the first few cycles as the pore throats are being bridged. When the calcite content exceeds 10%, the rock becomes brittle and can form plugs, which blocks the flow of solutions through the pores (DeJong et al., 2006; Zhu et al., 2024). A quantitative relationship is presented in Figure 2.

Literature in the EICP field had been reviewed systematically on the effects of temperature and particle size on treatment efficiency. A medium-grained sand (0.25 mm to 0.425 mm) was found to have the most highest UCS with EICP by Omarov et al. (2023); Fine sands had an uneven CaCO₃ distribution as a result of pore clogging and coarse sand had limited cementation as their pore size was too large for interparticle bonding. Jiang et al. (2025) found it to be true that the intrinsic specific energy of the EICP-treated medium sand samples was about 9,000 psi, while that of the coarse sand samples was 5,000 psi.

## 4 Engineering applications

MICP is an engineering technology that has a broad spectrum of potential engineering applications across very different sub-disciplines of civil and environmental engineering. The relative degree of maturity and impact in each of the principal domains is shown in Figure 2, and is discussed in detail in the following sections.

### 4.1 Ground improvement and soil stabilization

The first systematic study of the first major engineering application of MICP, ground improvement, began with DeJong et al. (2006), who showed increases in shear strength and stiffness in triaxial specimens of treated sand. Over the next ten years, the number of published column studies increased greatly and UCS values improved, ranging from early results of 0.1–0.5 MPa to over 10 MPa with optimized protocols (Whiffin et al., 2007; DeJong et al., 2010; Al Qabany et al., 2012). Whiffin et al. (2007) performed some of the first extended column tests specifically designed to assess upscaling, and found bacterial transport and distribution of calcite to be the primary constraints. Al Qabany et al. (2012) showed that multiple cycles of dilute treatment are more homogeneous in cementation than a single cycle of high concentration; this is relevant for the design of an injection strategy. A synthesis of the geotechnical properties of biocemented sands from dozens of studies was provided by Mujah et al. (2017).

According to the comprehensive statistical analysis of the treated sands with MICP, smaller d₁₀ values resulted in higher UCS values, with maximum UCS achieved at about 15% CaCO₃ content and low UCS values for CaCO₃ content greater than 15%. The dataset of 90 data points from 12 different sands shows UCS ranging from 0.06 MPa to 7.28 MPa, with sand properties (d₁₀: 0.09–0.60 mm, Cu: 1.03–10.10, Cc: 0.10–1.43) serving as boundary conditions (Hora et al., 2026). For a separate machine learning study with 243 specimens, the mean UCS was determined to be 1,328 ± 2,101 kPa (*n* = 243) (El-Sekelly et al., 2026). These reported maxima idicate isolated optima rather than replicable performance benchmark and must be interpreted with boundary conditions clearly stated, including soil properties (d₁₀, d₅₀, Cu, Cc, initial void ratio e₀), treatment protocol (urease activity in U/mLreagent concentrations, and number of treatment cycles), statistical descriptors (Mean + standard deviation with n ≥ 3 and test method standard), and test conditions (temperature, curing regime, and specimen aspect ratio).

One of the more recent reviews that was quite practically oriented was by Fu et al. (2023) which explicitly recognized the difference between laboratory and field performance and covered the range of ground improvement fundamentals to feasibility in the field. Their analysis supports the notion that the amount of calcite alone is not a complete indicator of field performance as the spatial distribution of the precipitate is also significant, specifically whether the calcite is at grain contacts or diffused over the grain surfaces, and this distribution is more challenging to control at scale (Fu et al., 2023; Chen et al., 2026). Quantitative evidence status for UCS and related strength outcomes are list in Table 4.

**Table 4:** Quantitative evidence status for UCS and related stength outcomes.

Source/Application	Matrix and conditions	Outcome	Mean ± SD (n)	Scale/Evidence	Interpretation
DeJong et al. (2006)	Sand; full treatment details require primary extraction	Strength/stiffness increased	Unavailable in the reviewed source set	Primary laboratory	Foundational proof; not a universal UCS value
Whiffin et al. (2007)	Columns; transport and distribution assessed	Soil improvement reported	Unavailable in the reviewed source set	Primary laboratory	Upscaling limited by bacterial/mineral distribution
Al Qabany et al. (2012)	Sand; dilute versus concentrated treatments	Uniformity differed by protocol	Unavailable in the reviewed source set	Primary laboratory	Protocol moderates spatial performance
Ahenkorah et al. (2024)	40 UCS tests across MICP/EICP and durability conditions	EICP showed lower wet–dry/freeze thaw resistance than MICP	Study total n = 40; subgroup means/SD not extracted	Primary laboratory	Durability comparison is condition-specific
van Paassen et al. (2010)	100 m^3^ treatment	Field scale stiffness response	SD and n unavailable in the reviewed source set	Primary pilot/field	Scale demonstrated; endpoint differs from UCS

Analytical interpretation. Stength outcomes cannot be ranked from isolated maxima because matrix, treatment shedule, curing, scale, and statistical reporting differ. The field demonstartion confirms scale and stiffness response, not transferability of a laboratory UCS benchmark.

### 4.2 Liquefaction mitigation

Because the benefit of *in situ* biocementation to withstand soil liquefaction under seismic loading is significant and because the other competing technologies (stone columns, cement grouting and compaction) have high environmental and/or logistics costs, some of the most intense MICP research has been initiated. In one of the first field-scale biostimulation tests for liquefaction resistance (Burbank et al., 2011), it was found that the liquefaction resistance of the indigenous ureolytic community could be stimulated in place to provide measurable increases in shear wave velocity; in a subsequent centrifuge test (Kamalzare et al., 2013), it was demonstrated that the liquefaction resistance of the specimens treated by the ureolytic community could be significantly greater than that of untreated control specimens when subject to repeated cycles of loading.

Denitrification was first discussed as an alternative pathway for liquefaction mitigation (O’Donnell et al., 2017), and the method is more practically viable at the target depths (usually 2–15 m) than ureolysis-based injection because it does not have the bacterial filtration limitation. The centrifuge work of DeJong’s group at UC Davis is the most mechanical of all available, and the authors have been very careful to emphasize the uncertainties in scaling from centrifuge to prototype scale (Dejong et al., 2014; Fu et al., 2023).

### 4.3 Erosion control and dust suppression

Surface erosion due to wind and water action and wind-blown dust from construction sites and arid land is an application that is well suited to MICP’s capabilities: the low-viscosity treatment solutions percolate into sandy surfaces by gravity without equipment or excavation, while the resulting calcite crust is mechanically resistant, and hydrologically transparent (compared to a cement-based surface sealer). Kavazanjian and Hamdan (2015) showed significant surface stabilization from wind erosion for field-scale loads of desert sand in Arizona. A 2025 Nature study has shown that a 346.67 kPa surface bearing capacity and 95% reduction in wind erosion occurred in a soil after three cycles of MICP, withstanding freeze–thaw and heavy rain impacts.

Ghasemi and Montoya (2022) expanded the erosion application to coastal sandy slopes and further developed a more permanent and ecologically sound protection system combining MICP treatment and the establishment of vegetation. They found that concentrations of ammonium greater than 50 mM after the treatment inhibited seedling germination, which is an important operational constraint as vegetation planting must be delayed until concentrations of ammonium have decreased to acceptable levels after a few weeks of natural dilution (Ghasemi and Montoya, 2022).

### 4.4 Self-healing concrete

One of the most commercially advanced MICP applications involves using dormant bacterial spores as a component of concrete to facilitate self-repair of cracks. The basic idea was introduced by Jonkers et al. (2010), who included spores of the bacteria *Bacillus* sp. in expanded clay particles embedded in concrete: the spores germinated when cracks formed and water entered the concrete, resulting in the precipitation of calcium carbonate (CaCO₃) which sealed the cracks. Van Tittelboom et al. (2010) compared the efficiency of microbial repair with chemical crack-sealing agents and determined that it is competitive in sealing efficiency up to about 0.5 mm crack width. This led to the development of more sophisticated methods to protect the bacteria’s survival during the highly alkaline concrete mixing process and enable them to remain viable over the life of the structure, first by silica gel then by hydrogel beads (Wang et al., 2012; Wang et al., 2014). *Bacillus* sp. Achal et al. (2011) demonstrated the potential to reduce the population of *B. thuringiensis*. The advantage of using CT-5 in cement mortar is to improve the compressive strength up to 36% without any crack sealing effect by enhancing the densification of the cement matrix.

**Figure 3:**
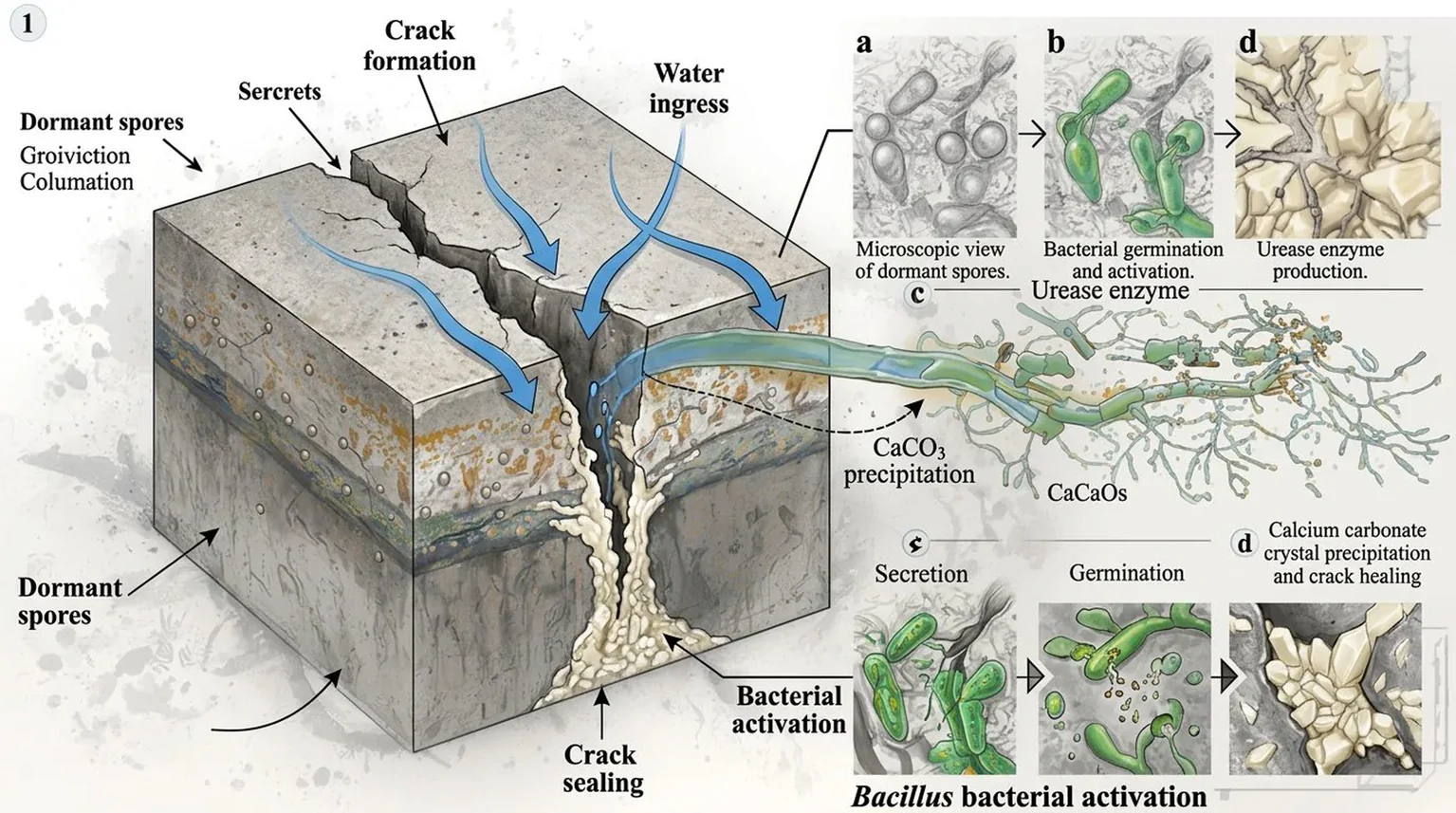
Self-healing concrete using MICP technology: The figure shows how embedded bacterial spores in concrete activate upon water ingress through cracks. The bacteria produce urease enzymes that trigger calcium carbonate precipitation, sealing the cracks autonomously. Inset panels **(a–d)** illustrate the process from dormant spores to bacterial activation, enzyme production, and crystal formation that repairs the damage.

This sealing has now been demonstrated in the laboratory for cracks up to 2.0 mm wide and the sealing provides nearly original impermeability. Omoregie et al. (2025) conducted a bibliometric and regulatory study of MICP in crack healing, revealing that regulatory requirements and standards are more limiting conditions for commercialization than technical performance. Self-healing mechanisms in the context of seismic and marine infrastructure were also reviewed by Jose et al. (2026), where they noted that biogenic healing presents some unique benefits where chemical capsule-based approaches either become ineffective or are inaccessible after damage to the marine infrastructure. However, the development of non-ureolytic self-healing systems by Omoregie et al. (2025) was a practically important innovation to prevent ammonia production in the concrete matrix using alkalitrophic bacteria (Figure 3).

The commercial viability of self-healing concrete has been supported by peer-reviewed studies evaluating product performance. Justo-Reinoso et al. (2023) conducted a comprehensive life cycle assessment of bacteria-based self-healing concrete, finding that while it has an 85% higher environmental footprint compared to conventional concrete in some metrics (primarily due to calcium nitrate and polyvinyl acetate production), strategic application can achieve sustainability improvements of 12–50%. The economic viability is enhanced by the ability to permit larger crack widths in design, potentially saving up to 51 kg CO₂ eq/m^3^. Xu and Sun (2026) demonstrated that microbial self-healing remains effective in aged concrete (up to 1 year), with 55–85% strength recovery and 81–99% permeability reduction across multiple product formulations.”

### 4.5 Heavy metal immobilization

Warren et al. (2001) and Fujita et al. (2004) laid the groundwork for understanding the mechanism of metal coprecipitation with biogenic calcite, demonstrating substitution of divalent elements with similar ionic radii into the calcite lattice during its growth. Fujita et al. (2004) specifically demonstarted strontium incorporation into biogenic calcite through isomorphous substitution, with distribution coefficients (0.5) significantly higher than those reported for abiotic calcite (0.02–0.4), estabilishing the fundamental mechanism by which divalent metals can be sequestered during calcite precipitation.

Bioremediation has been proven effective in removing heavy metals in several lab environments. For example, Kang et al. (2016) reported a consortium of four bacterial strains outperformed individual cultures in removing heavy metals from contaminated soils, achieving 98.3% lead and 85.4% cadmium removal within 48 h through MICP from contaminated soil matrices. The mixture exhibited synergistic benefits including enhanced urease activity, greater metal resistance, and increased calcite precipitation making it a more effective and sustainable bioremediation strategy for soils affected by mining and industrial activities. Kumari et al. (2016) thoroughly examined the mechanisms by which different heavy metals are immobilized by MICP and classified the immobilization mechanism of the various metals according to their ionic radii and precipitation chemistry.

Quantitative removal efficiencies must be interpreted with boundary conditions clearly stated. Zhuang et al. (2025) reported that *S. pasteurii* could remove Cd, Pb, Zn and Cr(III) by 95, 84, 97, and 98%, respectively, from contaminated solution, while from the aged refuse samples, the removal of exchangeable Cd, Pb and Zn were 74, 84 and 62%, respectively. The presence of Fe components from the old refuse, the Al components along with calcium carbonate precipitation, synergistically facilitated the conversion of the heavy metals to more stable and less bio-available form. In another study, *Viridibacillus arenosi B-21*, *Sporosarcina soli B-22*, *Enterobacter cloacae KJ-46*, *and E. cloacae KJ-47*, four different strains of bacteria isolated from abandoned Korean mine site, removed 98.3 and 85.4% of Pb and Cd from contaminated soils, respectively, within 48 h by synergistic MICP activity (Kang et al., 2016). This suggests that removal percentages measured in aqueous systems cannot be directly applied to soil matrices and that the performance of bacterial consortia is better than the performance of individual strains.

One of the most important factors that moderate the immobilization efficiency is metal toxicity on urease. Mugwar and Harbottle (2016) studied the effect of different heavy metals on urease activity and found that even at low concentration of 200 ppm, the urease activity was reduced up to 98% and due to inhibition of ureolysis it may be useful in the remediation of Cd at such concentrations. The intermediate inhibition was found for Zn^2+^ and Cd^2+^ while in case of Pb^2+^ the toxicity to ureolytic process is comparatively lower. The results confirm that metal toxicity of the enzymatic process is a key parameter in the efficiency of metal immobilization and that metal-specific toxicity should be taken into account in designing remediation strategies based on MICP.

*Sporosarcina pasteurii* was found to be more effective in immobilization of Cr(VI) in sand contaminated at 2000 mg/kg and 1,000 mg/kg with immobilization of 79 and 94% respectively, along with enhancing the UCS of sand treated specimens, as reported by Sharma and Satyam (2021). Their study also confirmed that bioprecipitation within the calcite lattice occurred to achieve long term stabilization and leachability tests showed that this immobilization method was effective. Oulad et al. (2025) reported that *Citrobacter* sp. *strain PO2* was isolated from petroleum-contaminated soil. The strain demonstrated efficient heavy metal removal via MICP at an initial concentration of 800 mg/L. Within 24 h, it achieved 99.89% Pb, 97.47% Zn, and 96.08% Cu removal. However, removal efficiency declined at higher concentrations. At 1200 mg/L, cadmium removal dropped to 22.07%. Nickel showed no significant removal above 800 mg/L. The metal toxicity ranking was established as Pb > Cu > Zn > Cd > Ni. This indicates that lead is most amenable to immobilization, while nickel is most inhibitory. MICP using this strain shows strong potential for remediating Pb, Zn, and Cu contamination. However, efficacy is highly dependent on both metal type and initial concentration (Oulad et al., 2025). Wang et al. (2023) conducted a comprehensive review on MICP-mediated heavy metal remediation, summarizing mineral structure of various heavy metal precipitates and factors influencing removal efficiency for Cu, Cd, Pb, Ni, Zn, Cr and mixed metals. The review also covered some drawbacks of the technique such as the influence of competing ions, soil matrix properties and necessity to carry out more long-term research on scale expansion and stability of the immobilized metals. Liu Y. et al. (2025) provided a systematic review of applications of MICP in the mining industry that include tailings solidification, heavy metal immobilization, use of mining wastes as resources, and carbon sequestration. They noted several challenges, such as the need to reduce microbial activity in extreme environmental conditions, low uniformity of calcium carbonate precipitation and a synergistic effect of metal toxicity plus low pH in acid mine drainage environments. The authors noted that these challenges must be addressed by holistic solutions that include MICP in combination with other remediation technologies to successfully implement the technology in the field. Evidence boundries for heavy metals immbolization are indiacted in Table 5.

**Table 5:** Evidence boundries for heavy metals immbolization.

Contaminant/Mechanism	Source	Key finding	Quantitative evidence	Matrix/Boundary conditions	Analytical insight
Sr (Strontium)	Fujita et al. (2004)	Sr^2+^ incorporated into calcite via isomorphous substitution during bacterial ureolysis	Distribution coefficient (Kd) = 0.5; significantly higher than abiotic calcites (0.02–0.4)	Ureolytic calcite precipitation system	mechanistic evidence for divalent metal sequestration
Mixed metals (general)	Warren et al. (2001)	Microbial calcite precipitation captures inorganic contaminants through coprecipitation	Qualitative mechanistic demonstration	Laboratory biomineralization system	Foundational study establishing coprecipitation mechanism
Mixed metals (review)	Kumari et al. (2016)	Comprehensive review of MICP mechanisms for toxic metal immobilization	Categorization based on ionic radii and precipitation chemistry	Review of multiple laboratory studies	mechanistic understanding
Mixed metals	Kang et al. (2016)	Bacterial mixtures containing ureolytic strains for heavy metal bioremediation	Qualitative demonstration	Bacterial consortia from Korean mine site	consortium approach over single strains; synergistic effects observed
Cu, Cd, Pb, Zn, Cr (review)	Wang et al. (2023)	Comprehensive review of MICP-mediated remediation for multiple heavy metals	Summarizes experimental results; no specific	Review of laboratory studies	general understanding;
Metal toxicity	Mugwar and Harbottle (2016)	Heavy metal toxicity to urease enzyme varies by metal type	Cu^2+^: up to 98% urease inhibition; Zn^2+^ and Cd^2+^: intermediate inhibition; Pb^2+^: lower toxicity	Defined metal concentrations; specific assay conditions	Critical finding: metal toxicity to urease is a primary constraint for MICP efficiency
Cd, Pb, Zn, Cr(III)	Zhuang et al. (2025)	*S. pasteurii* removes multiple metals via MICP	Solution: Cd 95%, Pb 84%, Zn 97%, Cr(III) 98%; Aged refuse: exchangeable Cd 74%, Pb 84%, Zn 62%	Solution: 0.5–2.0 mmol L^−1^; Aged refuse: spiked samples	Demonstrates matrix-dependent performance; soil efficiency lower than aqueous; Fe/Al components enhance stabilization
Cr(VI)	Sharma et al. (2025)	Cr(VI) immobilization in sand via bioprecipitation in calcite lattice	94% at 1000 mg/kg Cr(VI); 79% at 2000 mg/kg Cr(VI); UCS improvement also reported	Sand matrix; 1,000–2000 mg/kg contamination	Demonstrates MICP effectiveness for hexavalent chromium in sand.
Pb, Cd, Cu	Oulad et al. (2025)	MICP-based stabilization of heavy metal mixture by *Citrobacter* sp. strain PO2	Pb: 99.89%; Cu: 96.08% (at 800 mg/L inoculum); Cd: 22.07% (at 1200 mg/L)	Laboratory-optimized conditions; concentration-dependent	Removal efficiency highly dependent on initial metal concentration and inoculum size
Mining applications	Liu Y. et al. (2025)	Systematic review of MICP in mining: tailings, heavy metals, carbon sequestration	Qualitative review; identifies challenges: metal toxicity, low pH, heterogeneous substrates, microbial attenuation	Mining waste matrices; acid mine drainage conditions	Comprehensive review; emphasizes need for integrated approaches combining MICP with other technologies
Bacterial consortium	Kang et al. (2016) + supplementary data	*Viridibacillus arenosi* B-21, *S. pasteurii* B-22, *E. cloacae* KJ-46, *E. cloacae* KJ-47	Pb: 98.3%; Cd: 85.4% within 48 h; outperforms single strains	Contaminated soil from abandoned mine site	Synergistic effects: higher growth rate, urease activity, metal tolerance, calcite production

### 4.6 CO₂ sequestration

Geological carbon sequestration was cited by Krajewska (2018) as one of the possible high-value applications of urease-mediated calcite precipitation, since the mineral carbonate formed is thermodynamically very stable over geological time scales. Controlled batch experiments have reported carbon sequestration efficiencies up to 86.4%. The second application, but more relevant to the current projects, is for sealing of wells in the CO₂ storage formations and abandoned oil and gas wells, where biogenic calcite can plug the microchannels of the cement and annular voids that are leakage pathways (Krajewska, 2018). Phillips et al. (2018) (Section 6.1) presented the first field evidence of delivery and deposition of MICP at depths > 700 m via their DOE/NETL wellbore trials.

### 4.7 Mining and industrial waste applications

Liu Y. et al. (2025) made the first systematic review on the application of MICP in the mining industry, which included three different applications: solidification of tailings for slope stabilization and dust prevention; immobilization of heavy metals leaching from waste piles; and carbon sequestration using biocemented carbonate minerals. Pan et al. (2025) reported a novel use of MICP in wellbore drilling fluid for which the rheological properties of the MICP-treated bentonite muds are improved and prevent the collapse of the wellbore in difficult gravel strata. In order to utilize this high-volume industrial by-product, phosphoric acid manufacturing, as a road base material, Sun et al. (2026) studied the use of MICP treatment of phosphogypsum and obtained the target of strength and durability while satisfying the environment leaching requirement. All applications indicate that the role of MICP can be much more than that of traditional geotechnics and can be expanded to other fields of industrial waste valorization.

### 4.8 Coastal, offshore, and marine engineering

The low viscosity, biological compatibility and lack of construction traffic are values that are particularly beneficial in applications such as scour protection around offshore monopiles, stabilization of the seabed for submerged pipelines, and control of coastal slope erosion. The results of Peng et al. (2022) were encouraging for the use in marine applications as they showed that MICP treatment in seawater led to only about 2% reduction in CaCO₃ production compared to distilled water conditions. Laboratory and simulation tests have reported up to 100% decrease in scouring depth, and 1,743 kPa surface penetration resistance in model seabed sections. However, the application domain of the marine environment has not been fully developed yet, which includes challenges such as tidal forcing, seawater chemistry effects on carbonate equilibria, and the uncertain regulatory environment for the injection of biological material into the marine seabed; this domain should be seen at TRL 4–5 at present (Peng et al., 2022; Fu et al., 2023). Table 6 provides a consolidated summary of all engineering applications reviewed above.

**Table 6:** Summary of MICP enginering applications.

Application	Scale	Key quantitative results	Primary limitation	TRL	References
Ground improvement	Lab–Pilot	UCS 0.5–>10 MPa (protocol-dependent); permeability reduction 10–100×; treatment radius 1–3 m	Non-uniform CaCO₃ distribution at field scale; bacterial filtration limits penetration	6–7	Dejong et al. (2006), Whiffin et al. (2007), Al Qabany et al. (2012), Cheng et al. (2013), Mujah et al. (2017), Fu et al. (2023)
Liquefaction mitigation	Lab–Centrifuge	CRR improved; shear wave velocity (*Vs*) increase documented; increased resistance to cyclic loading	Uniform treatment at depth; NH₄^+^ by-product management; oxygen limitation	5–6	Burbank et al. (2011), Gomez et al. (2015), O’donnell et al. (2017)
Erosion/dust control	Lab–Field	Significant wind erosion reduction documented; surface crust formation; penetration resistance increase	Crust degradation under severe dry-wet cycling; rainfall erosion over time; NH₄^+^ inhibits vegetation establishment	6–7	Cheng et al. (2013), Kavazanjian and Hamdan (2015), Ghasemi and Montoya (2022)
Self-healing concrete	Lab–Commercial	Seals cracks ≤2.0 mm; 55–85% strength recovery; 81–99% permeability reduction; restores impermeability; 85% higher environmental footprint (LCA); up to 51 kg CO₂ eq/m^3^ potential savings	Long-term spore viability in commercial mixes; limited independent validation data; alkaline concrete matrix	8–9	Jonkers et al. (2010), Van Tittelboom et al. (2010), Achal et al. (2011), Wiktor and Jonkers (2011), Justo-Reinoso et al. (2023), Omoregie et al. (2025), Jose et al. (2026), Xu and Sun (2026)
Heavy metal immobilization	Lab	Pb: 94–99.2%; Cd: 96%; Zn > 93% (laboratory-optimized); removal efficiency matrix-dependent^2^	Cu toxicity inhibits urease (>98% inhibition); metal toxicity to bacteria; limited field validation	4–5	Warren et al. (2001), Fujita et al. (2004), Achal et al. (2013), Achal et al. (2011), Kang et al. (2016), Kumari et al. (2016), Mugwar and Harbottle (2016), Sharma et al. (2025)
CO₂/wellbore sealing	Lab–Field pilot	Permeability reduction demonstrated in thief zones at 310–700 m depth; CO₂ sequestration efficiency documented	Long-term stability under reservoir P–T conditions; deep-well delivery logistics; preferential flow	4–5	Krajewska (2018), Phillips et al. (2018)
Mining industrial waste	Lab	Tailings solidification; Pb leaching suppressed; slope stabilization demonstrated; dust prevention	Low pH + metal toxicity synergy; acid mine drainage conditions; heterogeneous substrate	3–5	Liu Y. et al. (2025), Pan et al. (2025), Sun et al. (2026)
Coastal/offshore	Lab–Simulation	Significant scour depth reduction documented; penetration resistance increase; seawater-compatible MICP demonstrated	Seawater chemistry effects on carbonate equilibria; regulatory gap; tidal forcing; uncertain permitting	4–5	Ghasemi and Montoya (2022), Peng et al. (2022)

^1^TRL follows the European Commission scale (1 = basic research, 9 = fully commercial). Values are consensus estimates based on published literature and may vary by specific implementation. ^2^Heavy metal removal percentages are reported from laboratory-optimized conditions with defined boundary conditions. Efficiency is highly dependent on initial metal concentration, matrix type (aqueous vs. soil), bacterial strain, and treatment duration. For example, Cd removal ranged from 61.1 to 89.3% in soil matrices (10–50 mg kg^−1^ Cd) compared to 99.6% in aqueous systems (Zhuang et al., 2025). Field-scale validation remains limited (TRL 4–5).^3^ UCS values for ground improvement represent the range from foundational laboratory studies. Field-scale tip resistance values (2–8 MPa within 12 cm radius) are more modest and site-dependent (Su et al., 2023; Hosseini et al., 2024).

### 4.9 Critical comparative analysis of MICP and ECIP performance

When quantitative results are compared across studies it is found that there is considerable variation in the superiority of the two types of soils depending on soil sample size and methods of soil treatment. There is no evidence to suggest that MICP and EICP are equivalent technologies, and that they can be used to replace each other.

#### 4.9.1 Strength performance: the particle size threshold

A machine-learning model developed from 517 experimental data points for MICP-treated sands suggests that the UCS of these soils is most strongly driven by the calcite content, and that soil properties such as the median grain size (D₅₀), coefficient of uniformity (Cu), and void ratio (e) are also significant factors in the strength response. The predictive accuracy of the ensemble models was good, using the metaheuristic algorithm, so the calcite content is not the only one that affects the UCS, but multiple factors interact for the determination of the mechanical behavior of treated soils (El-Sekelly et al., 2026).

When the sands treated with EICP were divided into various particle sizes, the results of parallel analysis showed that the sands that were medium-grained (0.25–0.425 mm) had the highest UCS and maximum velocity (Vmax) of the wave propagation, while the fine sands had uneven distribution of CaCO₃ due to the pore clogging, and the coarse sands had only limited cementation due to the oversized pores that hindered interparticle bonding (Omarov et al., 2023; Jiang et al., 2025). This sets a limit on the effectiveness of EICP based on particle size: medium sands give optimum pore filling and solution penetration. The intrinsic specific energy of the medium sand samples was found to be about 9,000 psi, while it was close to 5,000 psi for coarse sand samples, with treated samples showing some non-uniformity of strength (from + − 8,000 psi between the top and bottom halves) (Omarov et al., 2023).

#### 4.9.2 Biostimulation vs. bioaugmentation: a cost performance trade off

The results at the field scale confirm that biostimulation can be effective when correct carbon source doses are used, provided the urea hydrolysis is effective. The Bio-stimulation of loess soil from the Negev Desert resulted in a high rate of hydrolysis and alkaline pH and three to four sprayings per day were necessary for effective MICP (Raveh-Amit et al., 2024). Low yeast extract concentrations (0.1 g/L) were found to be inefficient for stimulation as compared to high concentrations (1.0 g/L) and thus a minimum threshold for stimulation efficacy was set. An additional benefit of biostimulation is the cost savings that result from the avoidance of the production, transport and homogeneous distribution of the ureolytic bacteria, which is an expensive and complex process that is difficult to achieve and may encounter regulatory obstacles. The use of naturally occurring ureolytic bacteria is a more feasible method to stimulate calcite precipitation in soils (Raveh-Amit et al., 2024).

#### 4.9.3 Heavy metals remediation: technology-specific window

When used in conjunction with other complementary technologies, MICP shows very metal-specific performance. In a study that investigated the synergistic effect of Corn stover biochar on contaminated soil and stabilization using electrokinetic remediation (EK) and microbial induced calcium precipitate (MICP), the urease activity was found to be significantly higher in the presence of corn stover biochar as compared to coconut shell biochar and bamboo biochar, with an increase of 49.4 and 25.4% respectively (Wang et al., 2025). The best sequence (EK treatment first followed by biochar-synergized MICP treatment) resulted in higher Cu stabilization efficiency of 51.90%, Cd stabilization efficiency of 73.40% and Pb stabilization efficiency of 36.26% than individual EK and MICP treatments. Energy consumption was reduced 12.16% compared to EK group and microstructural analysis showed that the microstructural analysis confirmed that the combined method made it easier to form stable calcite. The results of these studies all confirm that the combination technology is a very effective and environmentally friendly approach to decontaminate heavy metal polluted soil (Wang et al., 2025). The detailed of comrehensive comparison of MICP and ECIP performance across applications are provided in Table 7.

**Table 7:** Comprehensive comparison of MICP and ECIP performance across applications.

Application/Material	Performance metric	MICP result	EICP result	EICP-Lignin/Hybrid result	Optimal technology	References
Concrete crack repair	UCS Improvement	Notable improvement	Least effective	Most significant enhancement	EICP-Lignin	Xie et al. (2025)
Concrete crack repair	Flexural Strength	Moderate increase	Lower than MICP	Highest increase	EICP-Lignin	Xie et al. (2025)
Concrete crack repair	Calcium Carbonate Phase	Vaterite (predominant)	Vaterite (predominant)	Mixed vaterite + calcite	EICP-Lignin	Xie et al. (2025)
Concrete crack repair	Crack Sealing Capacity (≤0.35 mm)	Effective	Effective	Most effective	EICP-Lignin	Xie et al. (2025)
Sandstone heritage consolidation	UCS Increase	+15% hardness; +39% wave velocity	Lower than MICP	Not studied	MICP	Li et al. (2025)
Sandstone heritage consolidation	Reinforcement Depth	20 mm	Less than MICP	Not studied	MICP	Li et al. (2025)
Sandstone heritage consolidation	Color Compatibility (ΔE ≤ 5)	Conforms to acceptable threshold	Less compatible	Not studied	MICP	Li et al. (2025)
High-Density Soil (1.7 g/cm^3^)	UCS Increase	+26%	+8%	Not studied	MICP	Li et al. (2024)
High-density soil (1.7 g/cm^3^)	Wave Velocity Increase	+7.13%	+3.26%	Not studied	MICP	Li et al. (2024)
Low-density soil (1.5 g/cm^3^)	CaCO₃ Distribution	More uniform	Relatively consistent	Not studied	Comparable	Li et al. (2024)
Fine uniform sand	UCS Improvement (1 treatment)	178.4 ± 4.5 kPa (5.1 × control)	301.3 ± 9.7 kPa (8.6 × control)	Not studied	BEICP (bacterial enzyme)	Arshad et al. (2026)
Fine uniform sand	CaCO₃ Yield	9.8 mg/mL	11.6 mg/mL	Not studied	BEICP	Arshad et al. (2026)
Dolostone	UCS Improvement	+25%	+58%	Not studied	EICP	Ngoma and Kolawole (2024), Arshad et al. (2026)
Shale (with bedding)	UCS Improvement	−39% (weakening)	+1% (slight improvement)	Not studied	EICP	Ngoma and Kolawole (2024)
Shale (fractured)	UCS Improvement	+50%	Not reported	Not studied	MICP	Ngoma and Kolawole (2024)
Sand (equal CaCO₃ content)	UCS	Lower than EICP at same CaCO₃	Greater than MICP at same CaCO₃	Not studied	EICP	Ngoma and Kolawole (2024)
Sandstone (field trial)	Consolidation Effectiveness	Highly effective, short-term	Lower than MICP	Not studied	MICP	Li et al. (2025)
Cement-based materials	Crystal Structure Consistency	More consistent	Diverse crystal shapes	Mixed phase with calcite	MICP/EICP-Lignin	Jedrzejko et al. (2025)
Crack sealing (≤0.35 mm)	Sealing Capability	Effective	Effective	Most effective	All effective	Jedrzejko et al. (2025), Xie et al. (2025)
Durability (wet-dry/freeze–thaw)	Resistance	Superior to EICP	Lower resistance	Lower resistance	Not studied	Jedrzejko et al. (2025), Xie et al. (2025)

### 4.10 Laboratory to field performance gap

Literature analysis shows there is a significant and systematic gap between lab and field performance of the MICP tech (Chen et al., 2026). Laboratory test results have been consistently good with the UCS values increasing to more than 10 MPa, the removal of heavy metals in excess of 90% and the reduction of wind erosion up to 95% (Liu et al., 2021; Zhou et al., 2024). Value of these tests conducted at the field scale are much less consistent and lower. Laboratory indicators consist of erosion control and surface stabilization (92.6% soil loss reduction as confirmed by 11-month field erosion tests and the remarkable durability under natural rainfall conditions, realized by the optimized injection design, with tip resistance values of 2–3 MPa in a radius of 25 cm) (Liu B. et al., 2025). But there are still a number of achievements that are only laboratory-based: UCS > 10 MPa has not been validated in the field (tip resistance in the field is 2–8 MPa within 12 cm radius) (Liu B. et al., 2025), heavy metal removal >90% is only at TRL 4–5, with only limited field validation (maximum heavy metal removal rate 83% at a remediation depth of 20 cm and area of 1,000 m^2^ (Liu et al., 2021), a field study at an abandoned iron ore mine); and the durability under environmental stress, where there is significant degradation in field conditions. UCS exposed to the sea for 30 days had only 35.19% of its original strength, and the field marine conditions were the most severe factor, even compared to salt spraying, dry-wet cycles and temperature cycles alone (Li et al., 2023). The differences are caused by: (a) limited treatment radius (1–3 m) attributed to bacterial filtration and preferential flow (Sang et al., 2023); (b) the non-uniform distribution of CaCO3 as a result of the soil’s non-uniform characteristics (Li et al., 2023; Chen et al., 2026); (c) dissolution of CaCO3 due to environmental exposure; and (d) the disproportionate increase in scale-dependent heterogeneity at the scale of the field (Chen et al., 2026). They show that MICP can be an effective solution for erosion control and surface stability in the field, but the strength of the cementation and long-term durability still need to be proven in the field (Table 8).

**Table 8:** Field confirmed vs. laboratory only achievement in MICP.

Indicator	Laboratory achievement	Field confirmation	Status	Key reasons for discrepancy	References
Erosion control	92.6% soil loss reduction; 4.43 × penetration strength increase	50% evaporation reduction over 16 months; 11-month field trials confirmed durability under natural rainfall	Field-Confirmed	Laboratory conditions (controlled rainfall, uniform soil) vs. natural weathering (temperature fluctuations, saturation variation, salt transport)	Liu et al. (2025a)
Surface stability	Peak penetration strength increased 4.43 times	Surface strength of heavily cemented slope consistently higher than untreated; grass growth observed after 1 year	Field-Confirmed	Field slopes are significantly larger than models; manual spraying introduces variability	Zhang et al. (2024)
Injection homogeneity	Uniform CaCO₃ distribution in controlled columns	Achievable with optimized injection design; tip resistance: 2–3 MPa within 25 cm radius	Field-Confirmed	Reduced spacing between injection ports yields more evenly distributed peak resistance	Su et al. (2023)
High-strength cementation	UCS > 10 MPa (laboratory optimized)	Field tip resistance: 2–8 MPa within 12 cm radius; laboratory UCS values not replicated	Partially confirmed	Limited treatment radius (1–3 m) due to bacterial filtration and preferential flow; non-uniform CaCO₃ distribution from heterogeneous soil conditions	Su et al. (2023), Hosseini et al. (2024)
Heavy metal removal	>90% removal for Cu, Pb, Cd under optimized conditions	Maximum 83% removal at abandoned iron ore mine; remediation depth: 20 cm; area: 1000 m^2^	Lab-Only	Competition from indigenous microorganisms; heterogeneity in contaminant distribution; difficulty maintaining optimal pH and temperature *in situ*	Duarte-Nass et al. (2026)
Long-term durability (marine)	Resistance to temperature cycles demonstrated	After 30 days marine exposure: UCS dropped to 35.19% of initial strength; 6.69% mass loss	Lab-Only	Field marine conditions proved most severe deterioration factor—worse than salt spraying, dry-wet cycles, or temperature cycles alone	Li et al. (2023b)
Long-term durability (weathering)	Resistance to dry-wet cycles demonstrated	Over 16 months weathering: CaCO₃ decreased by >60%; evaporation rate reached nearly twice that of untreated soil	Lab-Only	Environmental exposure causes CaCO₃ dissolution; physical weathering (temperature fluctuations, salt crystallization) degrades crust	Zhang et al. (2024)
Long-term durability (freeze–thaw)	Laboratory suggests freeze–thaw resistance	Dry-wet cycles cause more significant deterioration than freeze–thaw (64.6% vs. 34.1% strength loss after 9 cycles)	Lab-Only	Soil particle size distribution and environmental exposure conditions differ significantly between lab and field	Li et al. (2023a)
Rainfall erosion (long-term)	Effective in short-term model tests	After 250 mm rainfall, erosion on treated slopes became more pronounced than untreated—performance degraded over time	Lab-Only	Variations in water content and temperature cause cracking in crust formed on slope surfaces; performance at long duration not as effective as model tests	Zhang et al. (2024)
Heavy metal stabilization	Cu removal 50–60% under all tested conditions	Field validation at TRL 4–5; limited field studies available	Lab-Only	Copper carbonate formation occurs independently of calcium ions; microbial ureolysis does not directly influence copper precipitation efficiency	Duarte-Nass et al. (2026)

## 5 Injection strategies and solution delivery

**Figure 4:**
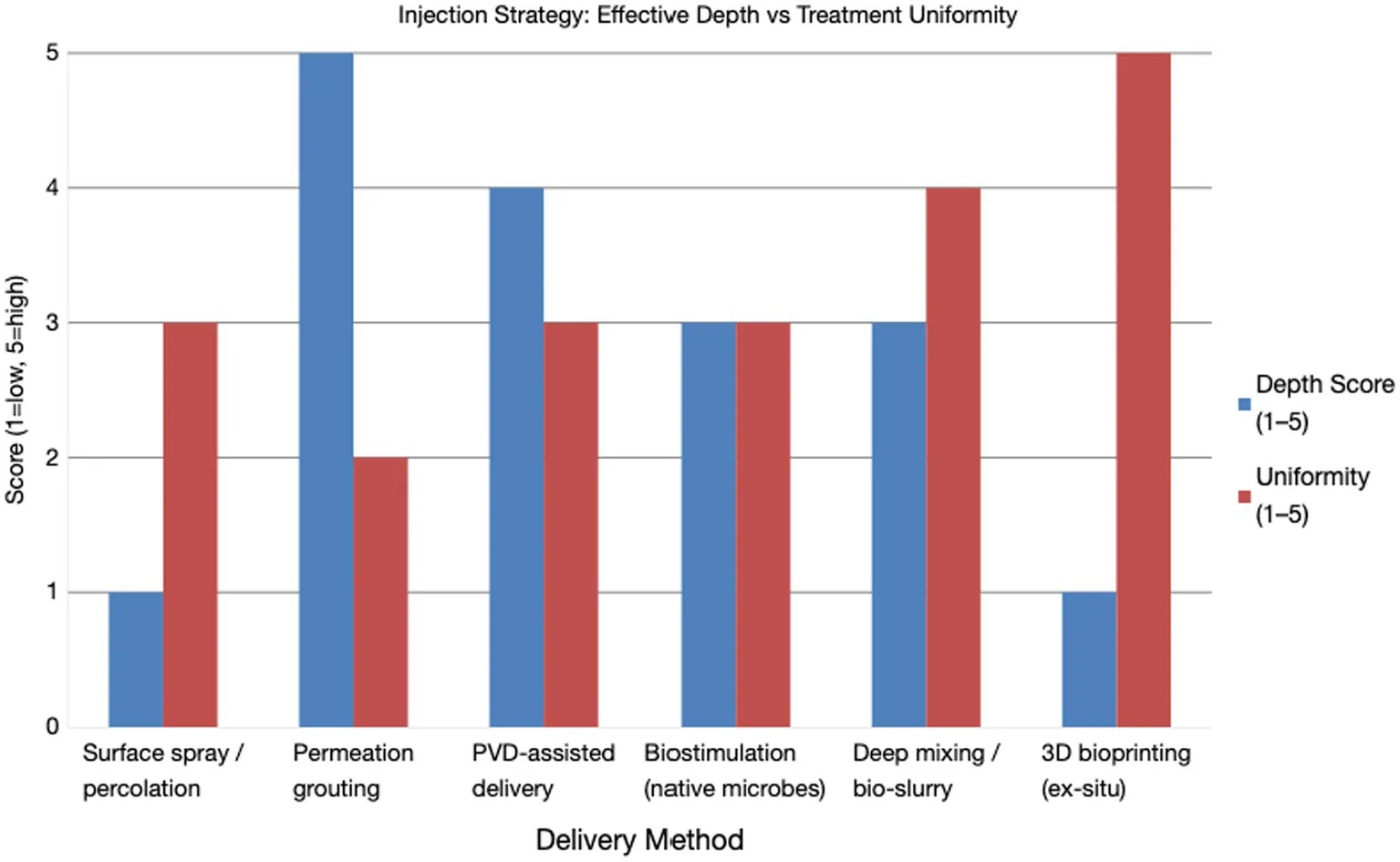
Comparative performance of major MICP injection and delivery strategies on a relative 1–5 scale for effective treatment depth and lateral uniformity. Scores are indicative and compiled from experimental and field data in the reviewed literature [10, (Whiffin et al., 2007; Cheng et al., 2013; Gomez et al., 2015; Kavazanjian and Hamdan, 2015; Ghasemi and Montoya, 2022; Raymond et al., 2025)].

How bacterial cells and cementation solutions are delivered to the target zone is decisive for both effectiveness and spatial uniformity. Four principal strategies have been studied, each with characteristic depth, uniformity, and cost profiles summarized in Figure 4 (Whiffin et al., 2007; Gomez et al., 2015; Kavazanjian and Hamdan, 2015; Ghasemi and Montoya, 2022; Raymond et al., 2025).

Surface spray/gravity percolation. Solutions are applied at the surface and allowed to percolate downward under gravity, creating a wide but shallow (0.3–1.0 m) treatment zone. Best suited to erosion control and dust suppression (Kavazanjian and Hamdan, 2015). Cheng et al. (2013) showed that this method achieves more uniform calcite distribution than injection grouting in unsaturated sands.

Permeation grouting. Low-pressure injection through boreholes or perforated pipes delivers solutions to depth. The standard strategy for geotechnical applications; used in all documented large-scale field trials (van Paassen et al., 2010; Kavazanjian and Hamdan, 2015). Principal limitation is preferential flow in heterogeneous soil, leading to over-treatment in coarse layers and under-treatment in finer ones (Mujah et al., 2017; Fu et al., 2023).

Prefabricated vertical drains (PVDs). Allow cementation solution to be delivered to discrete depth intervals while maintaining vertical drainage pathways for excess solution. Employed (Ghasemi and Montoya, 2022) in North Carolina coastal slope trials. Offers better depth targeting than surface application but adds installation cost.

Biostimulation of indigenous communities. Rather than injecting laboratory-cultured bacteria, this approach adds urea and calcium substrates to stimulate native ureolytic populations. Burbank et al. (2011) and Gomez et al. (2015) demonstrated its feasibility at field scale. Raymond et al. (2025) made the most comprehensive life-cycle argument for biostimulation, showing it is more environmentally sustainable than bioaugmentation in lifecycle assessment, mainly because it avoids the carbon and energy costs of ex situ bacterial cultivation and the risks of introducing non-native species. Post-treatment rinsing with approximately 1.8 pore volumes reduces NH₄^+^ to acceptable environmental levels in both approaches (Raymond et al., 2025).

## 6 Pathway to field-scale deployment

The progression from compelling laboratory results to reliable field application has been slower than early optimism suggested. This section reviews documented field experience, examines the practical barriers exposed, and discusses cost, environmental, and regulatory dimensions.

### 6.1 Documented field trials and pilot projects

**Figure 5:**
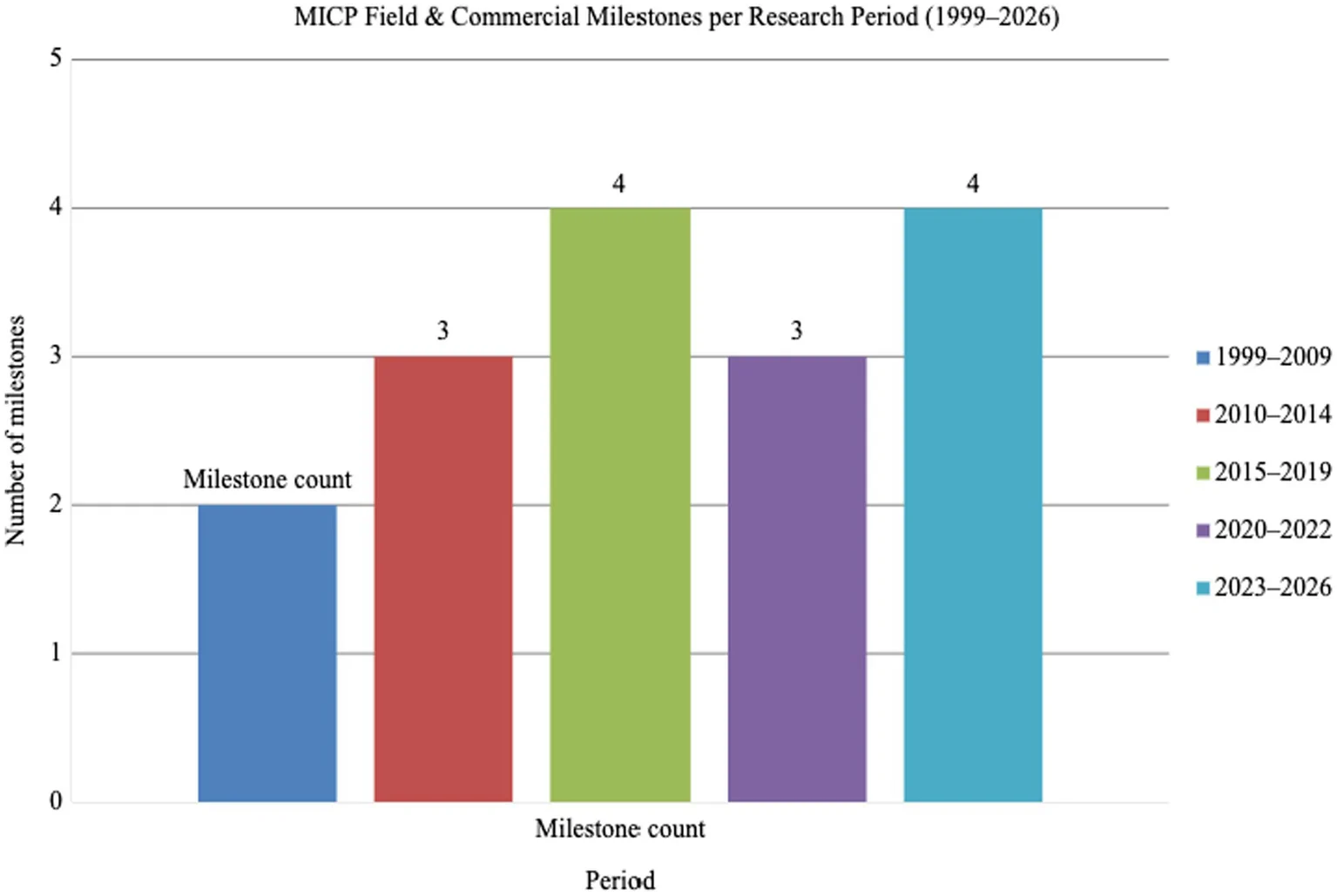
Selected milestones in field-scale and commercial MICP deployment (2010–2025). Each point represents a first-of-kind application or significant scale achievement. Data compiled from Van Paassen et al. (2010), Gomez et al. (2015), Kavazanjian and Hamdan (2015), Phillips et al. (2018), Lee et al. (2019), Esnault Filet et al. (2020), Ghasemi and Montoya (2022).

The seismic monitoring of a shear modulus increase from <20 MPa to ~350 MPa in the treated zone has been documented from the 100 m^3^ field trial in Papendrecht, Netherlands (2010) by van Paassen et al. (2010). This experiment has established the state-of-the-art for large-scale demonstration and revealed the central scale-up challenge – predicting reagent distribution and managing ammonium simultaneously at this scale. The DOE/NETL wellbore sealing trials in 2016 in Alabama and 2017 in Indiana successfully applied MICP to depths of 310–700 m and to a permeability reduction in ‘thief zones’ (van Paassen et al., 2010; Phillips et al., 2018). A field-scale biostimulation test conducted in the United States (Gomez et al., 2015) has documented that the indigenous ureolytic communities in a native sand site can be stimulated to increase measurable shear wave velocity. The first-ever successful field application of MICP to sand erosion control in desert areas, as reported by Kavazanjian and Hamdan (2015), was in Arizona. Ghasemi and Montoya (2022) reported a field-scale coastal slope erosion control trial in North Carolina that passed the challenges of hurricane, freeze–thaw and heavy rainfall events. Figure 5 shows the deployment timeline of these milestones.

Small-scale field demonstrations of biostimulation were conducted first to set the groundwork for larger demonstrations. Bacteriogenic mineral plugging in subsurface environments was first demonstrated by Ferris et al. (1996) and the injection and stimulation of ureolytic bacteria to precipitate CaCO₃ in the field was shown by Fujita et al. (2000). This was expanded by Cuthbert et al. (2013) who showed permeability reduction when fractured rock was biostimulated. Although the trials were smaller, on a scale of 100 m3, they demonstrated that biostimulation can be used as a field deployment method. Table S2 provide principal documented field trials and pilot-scale MICP deployments: analytical summary.

### 6.2 Scale-up challenges

There are a number of technical difficulties that repeatedly appear in the field trials considered. The most basic is treatment uniformity: preferential flow in a heterogeneous soil produces zones of over-treatment and untreated zones that are intrinsic to any of the injection-based techniques and more pronounced in the case of MICP as the biological reaction is spatially sensitive to the density of bacteria (Whiffin et al., 2007; Mujah et al., 2017; Fu et al., 2023). In the typical sand, the range of spatial extent from the injection point is limited to 1–3 m by the action of bacterial transport and filtration, which is not sufficient for many infrastructure purposes. Ammonium management is both a technical constraint as ureolysis is stoichiometrically required to produce 2 mol of NH₄^+^ for each mol CaCO₃, and a regulatory constraint because concentrations in the treatment area can reach 200–500 mM, which is well above the ecological toxicity thresholds. Lee et al. (2019) developed rinsing protocols that reduce post-treatment NH₄^+^ to acceptable levels, and explored biological nitrification as a secondary treatment, but neither has been validated at the volumes produced by a realistic large-scale project. Raymond et al. (2025) quantified that post-treatment rinsing with 1.8 pore volumes is effective but adds substantially to the water and energy balance of the treatment.

### 6.3 Cost–benefit analysis

One of the most important factors for the economic viability of MICP is the ability to use alternative reagents to the chemicals used in the laboratory. A low cost medium, based on baking soda, bakery yeast extract, snow-melting salt with soil fertilizer was able to reduce reagent costs up to 90% compared to standard ATCC formulations and still had a CaCO₃ content of 7.0–8.5% in treated mine tailings (Abharian and Sadrekarimi, 2026). Previously, field-scale slope stabilization in Japan has been carried out using media which is replaced with fertilizer urea, snow-melting agent, and beer-yeast, showing that the material cost is reduced about 97% (thirty-seven-fold) and a stiff surface layer is formed with strength of 0.14–1.02 Mpa (Gowthaman et al., 2023). Life Cycle Sustainability Assessment (LCSA) suggests that stimulating the indigenous microorganisms is more environmentally sustainable and cost-effective than bioaugmentation due to the need to avoid using the resources of bacteria cultivation and cold-chain logistics (Raymond et al., 2025). However, the effluent presents a major problem, but as high in NH4 + content as it is, post- treatment rinsing with ~1.8 pore volumes of rinse solution will reduce impacts to the environment; further optimization is required (Hiscott et al., 2025). Normalized to treatment performance, MICP can be cost competitive with traditional technologies, where the total treatment cost is estimated at $25–75/m^3^, and the costs associated with delivery are the largest contributors (Dejong et al., 2014).

## 7 Recent advances (2020–2026)

Research in MICP gained significant momentum following 2020, shifting the research focus from laboratory-based to commercialisation, process hybridisation, digital applications and the use of alternative microbial platforms. Bibliometric analysis of Xiao et al. (2025) revealed 5,373 publications on MICP, among them, amorphous calcium carbonate (ACC) and biofilm-mineral interfaces were identified as the hottest challenges in emerging research, indicating an ongoing maturation of the field from macroscale engineering demonstrations to microstructural mechanistic understanding.

### 7.1 Novel microorganisms and alternative pathways

Effective non-ureolytic viable MICP was demonstrated by Hemayati et al. (2023) using *Bacillus subtilis* and *Bacillus amyloliquefaciens* and calcium formate, which resulted in an average erosion control performance similar to ureolytic, but without generation of ammonium. Jain et al. (2021) have logically presented denitrification as the most field-friendly alternative to ureolysis for subsurface applications. Zhu et al. (2024) provide a preprint on the CRISPR-based genetic engineering platform of *S. pasteurii*, paving the way for rational engineering of urease activity and adding novel functions to genetically modified organisms for use in subsurface field trials, albeit with significant regulatory consequences. Chen et al. (2026) introduced a ‘Fine-Control MICP’ framework aimed at cross-scale geoenvironmental applications, offering a guideline for controlling the morphology, spatial distribution and life span of crystals simultaneously.

### 7.2 Hybrid and composite MICP techniques

Fiber-reinforced MICP addresses the inherent brittleness of pure biocemented soils by incorporating wool, polypropylene, or synthetic fibers to improve post-peak ductility and crack propagation resistance. Yan et al. (2025) demonstrated the synergy of modified biochar (MBC) with MICP for stabilizing Pb-contaminated loess: biochar provided nucleation sites, buffered lead toxicity, and improved shear strength by 52% while reducing permeability by 72% compared to MICP alone. Keykha et al. (2014) combined MICP with electro-osmosis to treat low-permeability soils that would otherwise be inaccessible to bacterial infiltration. These hybrid approaches collectively expand MICP’s practical application envelope beyond coarse sands toward the finer-grained materials that constitute a large fraction of real geotechnical problems.

### 7.3 Additive manufacturing and 3D bioprinting

Antorveza Paez et al. (2026) reported a 3D bioprinting process in which hydrogel-sand bio-inks inoculated with *S. pasteurii* were extruded layer-by-layer and subsequently treated with cementation solution to produce living mineral structures at decimeter scale, with compressive strengths of 1–4 MPa and demonstrated self-healing capability. The process addresses two challenges of conventional 3D concrete printing: inter-laminar bond weakness and dimensional shrinkage. Jose et al. (2026) identified 3D-printed bioconcrete as particularly promising for autonomous repair in seismic and marine structures. While the path from decimeter-scale demonstration to structural components is long, the conceptual foundation is established and the trajectory is clear.

### 7.4 Machine learning and data-driven optimization

Qiu et al. (2025) applied an ensemble machine learning framework (LARO-EnML) to predict UCS from MICP process parameters, achieving R^2^ = 0.957 and confirming calcite content as the dominant predictor. Ma et al. (2025) applied a CPO-CNN-LSTM hybrid model to predict shear strength in EICP/MICP-treated rubber-clay mixtures (R^2^ = 0.98), identifying 5% rubber content as optimal. El-Sekelly et al. (2026) used XGBoost with SHAP explainability tools for biocemented sand strength prediction, providing interpretable feature importance rankings directly usable by practitioners for protocol design. Collectively, these models allow exploration of the high-dimensional MICP parameter space urea concentration, calcium concentration, injection rate, bacterial density, treatment cycles, temperature, grain gradation that would require prohibitively many physical experiments to characterize by conventional methods. The proviso is that current models are trained on laboratory datasets, and transferability to field conditions with uncontrolled variables has not been established (El-Sekelly et al., 2026; Ma et al., 2025)

### 7.5 Commercialization status

There are evidence labeled commercial and government activities. The existence of Evonik’s current product page does not confirm its status as a self-healing agent for ready-mix and precast concrete, but rather it supports the product status, which is independent of its durability or service-life performance. A life-cycle study and boundary by a third party is required to validate BioMason sustainability statements as company claims. Biocalcis project information is commercial/grey evidence. DOE/NETL wellbore work is a government-supported test in the field, and not a commercial product. Table 9 provide the commercialization evidence and validation status as of 2026.

**Table 9:** Commercialization evidence and validation status as of 2026.

Technology/Organization	Application	Evidence type	Maximum validated status	Independent performance evidence	TRL	Analytical insight
Evonik SITREN® Selfheal 455	Concrete self-healing	Commercial/grey product page	Product exists; demonstrator projects ongoing	Company claims: up to 80% lower water absorption	8–9	Trend: Product is commercially available with claimed benefits (repeated crack healing, improved freeze–thaw resistance). Gap: Published results from demonstrator projects would strengthen the evidence base. Direction: Demonstrator projects are underway to generate real-world data.
Basilisk	Concrete admixture/repair	Commercial/grey	Product claims reported; field project documented (Rijen, Netherlands, 2025)	Independently validated in Rijen pump station project (TU Delft, SGS Intron) showing 10% lower environmental impact	8–9	Trend: Strongest independent validation among commercial products with real-world project data. Gap: Long-term monitoring still in progress. Direction: Independent research collaboration model provides a template for other commercial products to follow.
BioMason	Biocement materials	Commercial/grey	Company-reported production status	Company claims; independent validation limited	8	Trend: Established commercial-scale production. Gap: Independent peer-reviewed LCA data remains limited. Direction: Third-party certification would enhance credibility and market adoption.
Biocalcis®/Soletanche Bachy	Ground improvement	Commercial/grey/project report	Niche project deployment; BOREAL project (2019–2024); real-site applications documented	Peer-reviewed conference paper indicates 2–4% calcite yields significant erosion resistance	7–8	Trend: Most mature commercial geotechnical MICP application with a decade of project history. Gap: Peer-reviewed project metrics are incomplete. Direction: Publication of comprehensive case studies would enable broader industry adoption.
DOE/NETL-supported MICP	Wellbore sealing	Primary field/government program	Field demonstration: Phillips et al. (2018); 310–700 m depth; injectivity reduced	Peer-reviewed publication documents field performance in Gorgas #1 well; flow reduced from 0.29 m^3^/h to <0.011 m^3^/h	5	Trend: Public funding enabled deep-well demonstration at scales beyond private investment. Gap: Technology has not transitioned to commercial operation. Direction: Additional public-private partnerships could bridge the “Valley of Death” for this application.
Academic denitrification/formate/EICP	Alternative pathways	Primary laboratory/secondary	Laboratory-scale to limited pilot evidence	Application-specific; peer-reviewed laboratory studies available	3–4	Trend: Alternative pathways address ureolysis limitations (NH₄^+^ production, oxygen requirements). Gap: Most remain at laboratory scale; field validation is limited. Direction: Pathway selection should be guided by site-specific conditions.

### 7.6 Why commercialization pathways diverge

**Figure 6:**
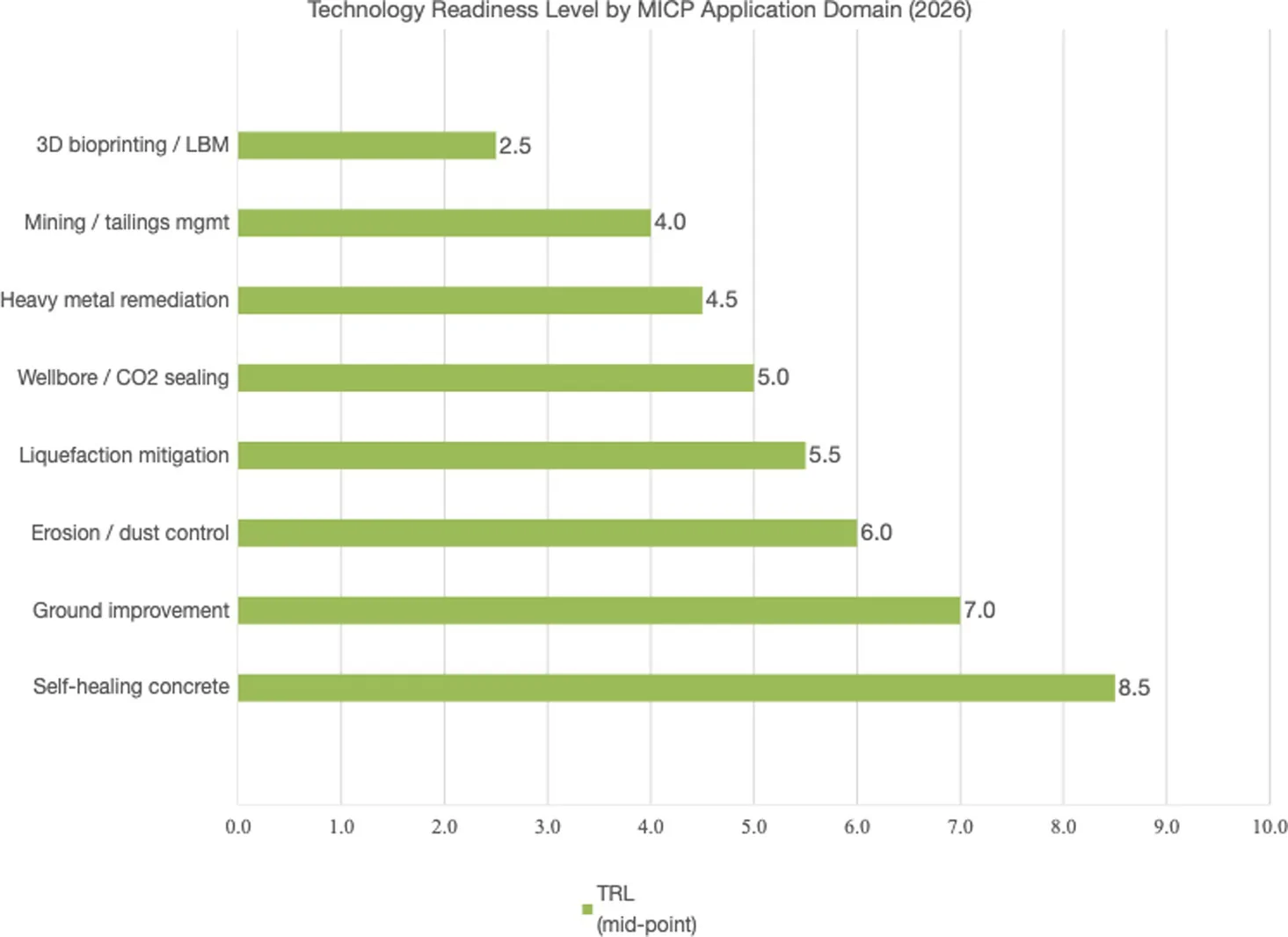
Technology readiness levels (TRL, European Commission scale) across MICP application domains as of 2026. Self-healing concrete has progressed furthest, with commercial products in active deployment. Geotechnical applications remain at TRL 5–7. Data synthesized from reviewed literature and commercial sources (Jonkers et al., 2010; van Paassen et al., 2010; Mujah et al., 2017; Omoregie et al., 2025; Raymond et al., 2025; Jose et al., 2026).

In comparison, self-healing concrete has progressed more quickly than subsurface MICP, since it is a bounded manufactured product: dose, carrier and curing can be controlled; the volume of the concrete treated is limited; there are established concrete testing methods; and a product specification can be imposed for liability. Geotechnical MICP needs to deal with heterogeneous geology, delivery to the subsurface, uncertainty in monitoring, logistics of nitrogen and water resources, permitting, and long-term liability of the site. Public funding has played important roles in field demonstrations like delivery with wellbores, while private industry and established materials companies can provide fundings for product formulation and market channels. No consensus, however, was found with respect to an open and harmonized break-even treatment volume, the distribution of the project-scale cost, or independently verified commercial performance. The two fields are reported instead of being estimated (Figure 6).

### 7.7 Analysis of commercial divergence: why self -healing concrete has ourpaced geotechnical MICP

The path to commercialization of MICP has been very different for the two major application areas: concrete repair and subsurface geotechnical. With commercial products from Basilisk, Evonik and BioMason, self-healing concrete is now at TRL 8–9, though geotechnical uses are still at TRL 5–7 (Son et al., 2024; Tumwiine et al., 2025). To be able to make realistic technology roadmapping and investment strategy, it is important to understand the structural factors that are causing this divergence.

#### 7.7.1 Technical and structural factors accelerating self healing concrete

Controlled Manufacturing Environment vs. Heterogeneous Subsurface. The self-healing concrete is a manufactured product and the factors such as bacterial encapsulation, curing conditions, carrier selection and dosing are controlled in a factory or batching plant. This controlled setting allows for consistent quality control, standardized testing methods, and consistent performance (Tumwiine et al., 2025; Wen et al., 2025). In contrast, geotechnical MICP has to deal with the variability of geology; the geology of natural soil deposits is unpredictable in terms of grain size distribution, mineral content, porosity and permeability (Son et al., 2024). Soil-specific transport and attached behavior of bacteria controls the uniformity and density of attached bacteria, which cannot be engineered away, and hence controls the uniformity of calcite. In all field trials even on relatively homogeneous material, preferential flow occurs resulting in over-treatment and under-treatment zones and the treatments radius cannot exceed 1–3 m due to bacterial filtration. This basic “heterogeneity” creates “design uncertainty” that is not found in concrete manufacture.

Self-healing concrete has also advanced over geotechnical MICP with a clear and quantifiable value proposition (such as 55–85% strength recovery, 81–99% permeability reduction, and bacteria viability in concrete matrix for more than 1 year), which directly correlates to available testing standards (such as ASTM and ISO) (Xu and Sun, 2026). Geotechnical benefits, such as liquefaction resistance and slope stability, on the other hand, are site-specific, and will not be demonstrated for decades after construction, and there are no standardized monitoring protocols. Product liability is also easier for self-healing concrete, because in this case, the manufacturer supplies a bounded admixture that is tested and verified against industry standards, but in geotechnical MICP, the design must be custom for each site and the manufacturer assumes no liability for the resulting concrete’s behavior in the subsurface. Additionally, the investment in self-healing concrete is lower than that of geotechnical MICP because it is an additive (10–30% of aggregate replacement), which is orders of magnitude lower than the costs associated with production of the equipment needed for large-scale cultivation, injection, water management, and ammonium remediation (Xu and Sun, 2026).

#### 7.7.2 Economic threshold between demonstration and commercial volumes

In the case of bacteria-based self-healing concrete, a life cycle assessment (LCA) revealed that a 1 m^3^ concrete sample has an 85% higher environmental footprint compared to conventional concrete, mainly due to the production of calcium nitrate and polyvinyl acetate, with 51% more water footprint (260 L) and 36% more embodied carbon footprint (120 kg CO₂ eq) (Justo-Reinoso et al., 2023). Sustainability improvements from 12 to 50% can be achieved, however, by selecting the use of bacteria-based self-healing concrete in specific areas of structures and taking advantage of its self-healing characteristics to permit larger crack width, up to 51 kg CO₂ eq/m^3^ can be saved (Justo-Reinoso et al., 2023). The long-term cost of producing one ton of coarse recycled concrete aggregate is about 40% lower than the cost of producing coarse natural aggregate and life cycle assessment has shown that the most cost-effective way of enhancing recycled aggregates is to use MICP (Zheng et al., 2025). There is no open literature published commercial project cost data for geotechnical MICP. A preliminary design guide for the field size injection strategy has been proposed, and efficiency factors have been proposed for well spacing and calcite content. A detailed techno-economic study for cultivation, reagents, injection, monitoring, water, ammonium treatment, and risk allocation for a reference project of 1,000 to 10,000 m^3^ has not been published, however (Son et al., 2024).

Geotechnical MICP - “Valley of Death”. The “Valley of Death” is a well-known issue in the field of innovation funding, defined by the distance between public funded research (TRL 4–5) and commercialisation (TRL 7–9). But this is the middle ground which is especially hard for the inventors to get funding — the space where invention must be converted to a product that will sell competitively. Grants and prizes are generally given at the R&D phase, whereas tax benefits and IP payoffs will only come in at the commercialization stage. Despite the basic tenet that innovation cannot survive if it is left to the free market alone, the “Valley of Death” is the period when startups must return to the free market in order to receive funding. The valley of death can occur at any point in the commercialisation process but can be particularly pronounced in the early stage/proof-of-concept of a company formation when there is a disconnect between the early stage nature of the technology and the start up of significant revenue generation and production. However, this is especially significant for geotechnical MICP, as a high level of investment is needed to develop large-scale demonstrators to validate the performance of the technology, which is beyond the means of friends, family and angel investors (Díaz-Catalán and Lobera, 2023).

#### 7.7.3 Distinct role of public vs. private funding

Public agencies have been vital in de-risking geotechnical MICP with large-scale field demonstrations that are too complex to be funded by a private sector, such as NSF-supported geotechnical treatment uniformity and biostimulation trials, and DOE/NETL wellbore sealing trials at 310–700 m depth. These were public investments that created the feasibility envelope that would be assessed by the private sector for commercialization. By contrast, private funding has been directed toward self-healing concrete, and Basilisk, Evonik and BioMason have built on existing supply chains and testing protocols to advance development by corporate R&D and venture capital investment (Omoregie et al., 2025). Geotechnical MICP is challenged by a special “Valley of Death” between the stages of public-funded research (TRL 4–5) and commercial viability (TRL 7–9) where funding for technology transfer is most limited. The gap exists because there is a need to spend more than what can be raised from early stage investors on the large scale demonstrators requested by the geotechnical applications sector, and there is a lack of long-term performance data for adoption by the construction industry decision makers (Fahimizadeh et al., 2022; Omoregie et al., 2025). The commercial gap will continue to inhibit geotechnical MICP deployment if it is not supported by public funds for field trials and standardized protocol development.

## 8 Research gaps and future directions

An extensive literature review for this study repeatedly indicates six critical gaps that, if not resolved, will significantly hinder the widespread adoption of MICP in engineering practice. They are not simply issues of academic interest, but all real deployment challenges: management of the ammonium by-products on an operational scale, lack of long-term durability data under realistic loading and environmental conditions, inherent difficulty treating fine-grained soils, absence of standardized monitoring and certification protocols, need for full-cycle techno-economic analysis (TEA), and under-exploitation of indigenous microbiomes through biostimulation.

These challenges can only be met through a coordinated, systematic and targeted approach, driven by data. We suggest a time-frame of one to three years to build the evidence base, building on existing measurement frameworks. The first 12 months will be focused on getting past one-off instances of maximum performance and finding a way to develop a culture of reproducible and transparent reporting. This means that all MICP experiments should have a minimum reporting dataset. The key factors are a strict mass balance for ammonium and a goal to close the mass balance for ≥90% of the input nitrogen in bench scale and column tests using conventional techniques such as ion chromatography. At the same time, a default method of specimen preparation and testing should be published that would enable cross-study comparisons, especially for durability testing against wet-dry, freeze–thaw, and cyclic loading methods based on ASTM or ISO standards. The first year should be dedicated to a community consensus on a reference functional unit, currency year and system boundary for a reference project as required for a crucial task: techno-economic assessment (TEA) following the principles of ISO 14040/14044 (Life Cycle Assessment (LCA)). This will give the framework to carry out a meaningful Monte Carlo sensitivity analysis of the most influential cost drivers.

The second year (months 12–24) should be reserved for pilot scale validation and inter-laboratory collaboration based on this foundational information. The most important goals are: (1) to conduct an “around the clock” study in which multiple laboratories perform durability tests on biocements to quantify uncertainty and variability under standard conditions, (2) to test a pilot ammonium recovery/treatment system that provides a complete water and energy balance, to demonstrate effectiveness in terms of reduction of residual ammonium levels to ecologically acceptable levels and residues, (3) to release an auditable, comprehensive cost inventory database for a pilot ground improvement project, to enable a preliminary but rigorous comparison of the costs of biocement with conventional methods, and (4) to pilot controlled biostimulation in different soil environments, for which metagenomic characterization of the site will inform the processes and effectiveness of the biostimulation and containment of native ureolytic communities. For the ongoing issue of fine-grained soils, this should be dedicated to establishing a clear ‘transportability window’ for all the available alternative delivery options such as EICP, biopolymer or electrokinetic-assisted treatment methods, for at least three soil classes, e.g., a silty sand, a clayey silt and a sandy clay.

At the 36-month point the body of evidence should be large enough to enable the development of clear go/no-go criteria. A pathway to deployment is possible only if the following is true: (a) a site-specific discharge/reuse criterion can be met with a demonstrated energy and water-balanced recovery system; (b) an application-specific durability retention criterion is met and verified by the multi-laboratory round-robin study; (c) a delivery strategy for fine-grained soils results in a desired uniformity and mechanical performance, while avoiding generation of undesirable by-products; and (d) the techno-economic assessment indicates a competitive application under a declared scenario and system boundary. Perhaps importantly, the 36 month period of monitoring is not a full-fledged certification standard, but rather a draft performance-based protocol that includes tools such as shear wave velocity (*Vs*), Cone Penetration Testing (CPT) and calcite/carbonate mapping. A blind comparison to destructive tests is necessary to demonstrate the validity of this protocol, and should provide a scientific rationale for acceptance for regulatory purposes. This roadmap is not a one-size-fits-all or a promise of a universal price target, but rather a structured framework to develop the auditable, high quality evidence practitioners, investors and regulators are seeking to enable them to embrace MICP wholeheartedly.

## 9 Conclusion

Whole-cell kinetics, nutrient limitation, transport, and ammonium management are design critical and ureolytic MICP is the most well-studied and field proven pathway. Wherever living cells are used, whole-cell parameters should be used in place of the purified-enzyme assumptions. Second, laboratory studies indicate potential for ground improvement, erosion control, crack healing, and contaminant immobilization that is significant; but there are many headline outcomes for which the means, SDs, n and boundary conditions are not consistently extractable. These successes are isolated and not across the board. Third, field evidence confirms delivery and selected outcomes at demonstration scale including ~100 m3 ground treatment, field biostimulation, coastal-slope treatment and deep-well sealing. It is not yet suitable to move maxima of three types of UCS (transferred from the laboratory) to heterogeneous locations, such as removal or durability. The major industrial barriers are uniformity, logistics (including nitrogen and water), monitoring, long-term performance, and project economics. Fourth, and most obviously, the self-healing concrete (SHC) has, by itself, the clearest product pathway, as both manufacture and quality assurance are constrained, while the subsurface applications involve site design and permitting. The next 1–3 years have the highest priorities for an auditable minimum dataset, replicated durability and monitoring protocols, closed nitrogen balances, reference-project techno-economics/LCA, and field pilots with explicit go/no-go criteria. These steps would determine if application-specific performance can be replicated at reasonable environmental and economic expense or not.
