# Supplementary material for: Microbially induced calcite precipitation (MICP): mechanisms, engineering applications, and pathway to field-scale deployment — a comprehensive review
Source: Front Microbiol. 2026 Aug 27;17:1928040. doi: 10.3389/fmicb.2026.1928040 (PMC13559790; doi:10.3389/fmicb.2026.1928040)
Supplement: Supplementary file 1 [file Table_1.docx]

**Supplementary Tables**

**Table S1: Mechanisms to enginering design map**

| **Mechanism/evidence** | **Quantitative observation** | **Engineering variable** | **Design implication/limitation** |
| --- | --- | --- | --- |
| Whole-cell urea kinetics; (Lauchnor et al., 2015) | Km 305 mmol L⁻¹; first-order k1 0.35 h⁻¹ up to 330 mmol L⁻¹ | Residence time; pulse concentration; injection interval | Calibrate with whole cells under site chemistry; do not import purified-enzyme kinetics |
| Cell-density dependence;  (Lauchnor et al., 2015) | Linear over 1×10⁷–2×10⁸ CFU mL⁻¹ | Flow rate; retention; treatment radius | Couple reaction rate to measured retained cell density and transport |
| NH₄⁺/pH response; (Lauchnor et al., 2015) | No rate effect up to 0.19 M NH₄⁺ or pH 6–9 in the tested batch system | Pulse chemistry; rinsing interval | Not a universal field threshold; nitrogen management remains stoichiometric |
| Auxotrophy; (Lapierre et al., 2020) | Four nutrient requirements identified | In situ biostimulation vs ex situ cultivation | Confirm site nutrient limitation before selecting inoculation strategy |
| Double operons; (Pei et al., 2025) | Structure demonstrated; field control rule not reported | Culture control; expression monitoring | Use expression data as a research tool, not as a field design coefficient |
| Transport and pore-throat filtering | Study- and site-specific | Well spacing; injection pressure; treatment radius | No universal well spacing can be supported from the present evidence |

Note:Analytical interpretation. Whole cell kinetics, retained biomass, nutient requirements, and transport jointly govern tratment sheduling. These mechanisms constrain design choices but do not yeild a universal injection intercal, treatment radius, or well spacing.

| **Study** | **Location** | **Scale** | **Year** | **Key Outcome** | **Key Challenge** | **Analytical Insight** |
| --- | --- | --- | --- | --- | --- | --- |
| Bio-Grout large-scale | Papendrecht, NL | 100 m³ | 2010 | Shear modulus → 350 MPa; seismic monitoring confirmed | Reagent uniformity; NH₄⁺ management | Trend: Established the state-of-the-art for large-scale demonstration; revealed that predicting reagent distribution at scale is the central challenge. Direction: Multi-well injection strategies with 1 m spacing are needed for uniform treatment (Sang et al., 2023). |
| Field biostimulation test | USA | ~200 m² | 2011 | Vs increase via native ureolytic stimulation | Spatial variability; monitoring resolution | Trend: First evidence that indigenous ureolytic communities can be stimulated in place without exogenous bacterial injection, reducing cultivation costs. Direction: Site-specific nutrient optimization and real-time monitoring are critical for success. |
| Arizona wind erosion control | Arizona, USA | Field scale | 2014–15 | Measurable surface stabilization against wind loads | Crust durability under rain | Trend: Demonstrated MICP viability for surface applications where shallow treatment (0.3–1.0 m) is sufficient. Direction: Long-term crust durability under environmental exposure requires further study; biostimulation may be more sustainable than bioaugmentation for erosion control (Raymond et al., 2025). |
| Biostimulation field trial | USA | Field scale | 2015 | Vs increase; soil resistance improved | Shallow depth limitation | Trend: Confirmed biostimulation effectiveness for shear wave velocity improvement but limited to shallow depths (<3 m) due to reagent penetration. Direction: Combining biostimulation with low-pressure injection and PVDs may extend treatment depth (Ghasemi and Montoya, 2022). |
| Gorgas #1 wellbore | Alabama, USA | 310 m depth | 2016 | Injectivity reduced; USIT confirmed solids | Deep-well delivery logistics | Trend: First successful MICP application at significant depth; demonstrated that bacterial transport and retention are feasible under deep-well conditions. Direction: Deep-well applications require careful attention to in situ temperature, pressure, and geochemistry (Phillips et al., 2018). |
| Rexing #4 wellbore | Indiana, USA | ~700 m depth | 2017 | Thief-zone permeability reduced; pressure restored | Preferential flow; hurricane delay | Trend: Showed that MICP can seal targeted thief zones even at extreme depths; pressure restoration confirmed permeability reduction. Direction: Field conditions (e.g., weather delays) introduce uncertainty that laboratory studies cannot capture; project planning must include contingencies. |
| Coastal sandy slope | N. Carolina, USA | Field slope | 2020–22 | Hurricane/freeze-thaw survival; erosion reduced | NH₄⁺ inhibited seedling germination | Trend: Most comprehensive coastal field trial; demonstrated MICP resilience to extreme weather events. Direction: NH₄⁺ concentrations >50 mM inhibit vegetation; post-treatment rinsing (1.8 pore volumes) or planting delays are required for ecological compatibility (Ghasemi and Montoya, 2022; Raymond et al., 2025). |
| Biocalcis® reinforcement | Europe (Italy+) | Commercial | 2011–present | Maritime structures & embankments reinforced | Industrial cost vs. chemical grouts | Trend: Commercial niche established for high-value applications where chemical grouts are unsuitable; underscores that MICP is not a universal replacement but a specialized alternative. Direction: Cost reduction through biostimulation and industrial-grade reagents is needed to compete with conventional methods (Raymond et al., 2025). |

**Table S2.** Principal documented field trials and pilot-scale MICP deployments: analytical summary.

**Notes:**

¹ Outcomes are based on published accounts; some commercial data may not be fully disclosed.

² TRL assessment for field trials: Bio-Grout (TRL 6–7), Wellbore sealing (TRL 5), Coastal slope (TRL 6), Erosion control (TRL 6–7), Biocalcis® (TRL 7–8).

³ Key analytical insights are synthesized from the reviewed literature and are intended to highlight cross-cutting trends rather than provide definitive conclusions for every field scenario.
